# Revitalizing Photoaging Skin through Eugenol in UVB-Exposed Hairless Mice: Mechanistic Insights from Integrated Multi-Omics

**DOI:** 10.3390/antiox13020168

**Published:** 2024-01-29

**Authors:** Tao Tong, Ruixuan Geng, Seong-Gook Kang, Xiaomin Li, Kunlun Huang

**Affiliations:** 1 Key Laboratory of Precision Nutrition and Food Quality, Key Laboratory of Functional Dairy, Ministry of Education, College of Food Science and Nutritional Engineering, China Agricultural University, Beijing 100083, China; bs20213060550@cau.edu.cn (R.G.); foodsafety66@cau.edu.cn (K.H.); 2 Key Laboratory of Safety Assessment of Genetically Modified Organism (Food Safety), Ministry of Agriculture, Beijing 100083, China; 3 Beijing Laboratory for Food Quality and Safety, Beijing 100083, China; 4 Department of Food Engineering and Solar Salt Research Center, Mokpo National University, Muangun 58554, Republic of Korea; sgkang@mokpo.ac.kr; 5 Institute of Quality Standard and Testing Technology for Agro-Products, The Chinese Academy of Agricultural Sciences, Beijing 100081, China; lixiaomin@caas.cn

**Keywords:** UVB exposure, photoaging, eugenol, multi-omics, transcriptome profile, gut microbiota

## Abstract

Chronic ultraviolet (UV) exposure causes photoaging, which is primarily responsible for skin damage. Nutritional intervention is a viable strategy for preventing and treating skin photoaging. Eugenol (EU) presents anti-inflammatory and antioxidant properties, promotes wound healing, and provides contact dermatitis relief. This study explored the ability of EU to mitigate skin photoaging caused by UVB exposure in vitro and in vivo. EU alleviated UVB-induced skin photodamage in skin cells, including oxidative stress damage and extracellular matrix (ECM) decline. Dietary EU alleviated skin photoaging by promoting skin barrier repair, facilitating skin tissue regeneration, and modulating the skin microenvironment in photoaged mice. The transcriptome sequencing results revealed that EU changed the skin gene expression profiles. Subsequent pathway enrichment analyses indicated that EU might reverse the pivotal ECM–receptor interaction and cytokine–cytokine receptor interaction signaling pathways. Furthermore, EU alleviated the intestinal dysbiosis induced by chronic UVB exposure. Spearman analysis results further revealed the close connection between gut microbiota and skin photoaging. Considering the near-inevitable UVB exposure in modern living, the findings showed that the EU effectively reverted skin photoaging, offering a potential strategy for addressing extrinsic skin aging.

## 1. Introduction

The skin, being the body’s largest organ, commonly exhibits signs of aging as part of the overall aging process [1]. More importantly, skin aging impacts whole-body aging. Aging and cellular senescence phenotypes in the skin were found to correlate with immunosenescence, longevity, or cardiovascular disease risk [2]. The skin aging process is influenced by two primary factors: intrinsic factors, including genetic makeup, somatic capacity, and composition, and extrinsic factors, such as environmental influences, nutrition, and lifestyle [3]. Of these, UVB-induced skin photoaging is considered the primary factor responsible for skin damage, accelerating skin aging. For decades, the term photoaging has been used synonymously with extrinsic skin aging, underscoring the significance of solar UV radiation [3]. Skin photoaging is characterized by macroscopic symptoms such as dryness, roughness, sagging, deep wrinkles, excessive pigmentation, and vasodilation. In severe cases, it can lead to benign or malignant tumor development, including malignant melanoma, squamous cell carcinoma, and solar keratosis [4,5]. Molecular-level studies have shown that UVB radiation decreases the extracellular matrix (ECM) levels and matrix metalloproteinase (MMPs) accumulation, upregulates senescence-associated secretory phenotypes (SASPs), and increases the p21 senescence marker and p53 DNA damage marker levels in the skin [2,6].

Various approaches are employed to prevent or address skin photoaging, including physical protection methods such as sunglasses, window films, clothing, topical treatments with active ingredients, and medical cosmetology [7]. The use of dietary interventions to improve photoaged skin has gained popularity recently due to increased health consciousness and higher quality of life. Both animal and clinical studies suggest that incorporating functional compounds such as phytochemicals, vitamins, probiotics, functional sugars, and oils into the human diet can effectively protect the skin against photoaging damage [8,9].

Eugenol (EU), also known as 4-allyl-2-methoxyphenol, is a colorless to yellow liquid with a strong clove odor [10]. It occurs naturally in various spices, such as cloves, basil, cinnamon bark, and turmeric [11], and is also the main component of clove essential oil [12]. EU is commonly used as a local anesthetic and an analgesic in dentistry [13], while the Food Safety National Standard for the Use of Food Additives (GB2760-2014) by the National Health and Family Planning Commission of China also lists it as an authorized food flavor [13]. EU exhibits several biological activities, including anesthetic, analgesic, anti-inflammatory, antimicrobial, antioxidant, and anticancer properties [14]. Our previous study demonstrated that dietary EU mitigated adiposity and regulated gut microbiota in mice fed a high-fat diet [15]. It also offers various skin benefits, including promoting wound healing [16], alleviating contact dermatitis [17], inhibiting skin tumors [18], and enhancing the skin permeation of drugs [19]. Hwang et al. reported that 50% ethanol extract of clove exhibited anti-skin photoaging effects in vitro and in vivo, and one of its main components, EU, has been shown to mitigate dermal fibroblast photoaging in vitro [20]. In summary, EU exhibits significant potential in preventing skin photoaging induced by chronic UVB exposure in mice. This study explores the ameliorating effect of dietary EU on chronic UVB-induced skin photoaging and integrates insights from skin transcriptomics and microbial communities to reveal the underlying mechanisms.

## 2. Materials and Methods

### 2.1. Materials

The EU (99% pure) was obtained from Sigma (St. Louis, MO, USA), while the TRIzol was sourced from Invitrogen Life Technologies (Carlsbad, CA, USA). The reverse transcription and SYBR Green Supermix kits were procured from Tiangen Biotech (Tiangen, Beijing, China), while the paraformaldehyde fixative was purchased from Servicebio Biotechnology Company (Wuhan, China). The Coomassie Brilliant Blue G250 (C8420) and phosphate-buffered saline (PBS, P1022) were obtained from Solarbio Biotechnology Company (Beijing, China). The Dulbecco’s Modified Eagle Medium (DMEM, D0822) was sourced from Sigma (St. Louis, MO, USA), while the Modified Eagle Medium (MEM, C11095500BT), fetal bovine serum (FBS, 10270-106), penicillin-streptomycin solution (15140-122), and 0.25% trypsin-ethylene diamine tetraacetic acid solution (25200-056) were obtained from Gibco (Thermo Fisher Scientific, New York, NY, USA). The Cell Counting Kit (CCK)-8 (C0037), Reactive Oxygen Species (ROS) Assay Kit (S0033S), Malondialdehyde (MDA) Assay Kit (S0131S), and Superoxide Dismutase (SOD) Assay Kit (S0101S) were procured from Beyotime Biotechnology Company (Shanghai, China). The Human Total MMP-1 Enzyme-Linked Immunosorbent Assay (ELISA) Kit (DY008) and the Human Hyaluronic Acid (HA) ELISA Kit (DHYAL0) were provided by R&D Systems (Minneapolis, MN, USA). The Procollagen Type I C-peptide (PIP) ELISA Kit (MK101) was purchased from Takara Bio (Kusatsu, Japan), while Silflo silicone was obtained from Jinhongfan Trade Co. (Beijing, China). The mouse HA ELISA Kit was provided by Cusabio Technology Company (Beijing, China).

### 2.2. Cell Culturing and UVB Irradiation

The HaCaT cells, a human epidermal keratinocyte cell line, were procured from the Cell Resource Center of Peking Union Medical College (Beijing, China). The Hs68 cells, a human dermal fibroblast cell line, were sourced from the American Type Culture Collection (Manassas, VA, USA). The HaCaT cells were cultured in MEM supplemented with a 10% FBS and 1% penicillin-streptomycin solution, while the Hs68 cells were maintained in DMEM supplemented with a 10% FBS and 1% penicillin-streptomycin solution. Both cell lines were incubated in a 5% CO_2_ atmosphere at 37 °C. The cells were washed with PBS for subculturing, while a 0.25% trypsin/ethylenediaminetetraacetic acid solution was used for detachment.

A UVB lamp (TL 20W/12 RS SLV/25, Philips, Amsterdam, The Netherlands) was set at a fixed distance of 30 cm from the cells to provide UVB irradiation. An irradiation intensity of 0.126 mW/cm^2^ was measured using a radiometer (Beijing Normal University Optical Instrument Factory, Beijing, China) equipped with a UVB sensor capable of detecting UVB (with a peak at 297 nm). The doses were calculated using Equation (1):

(1) $$\mathrm{Irradiation}\;\mathrm{time}\;\left(s\right)=\mathrm{irradiation}\;\mathrm{dose}\;\left({\mathrm{mJ}/\mathrm{cm}}^{2}\right)\;\text{÷}\;\mathrm{irradiation}\;\mathrm{intensity}\;\left({\mathrm{mW}/\mathrm{cm}}^{2}\right)$$

This equation was used to determine the required irradiation time for the desired UVB dose. The cells were kept in 50 μL PBS throughout the irradiation process.

### 2.3. Cell Viability Assay

A CCK8 assay was used to determine the viability of the cells, which were seeded into 96-well plates and cultured for 24 h. Each well was treated with either the vehicle (DMSO) or EU and incubated for another 24 h. The blank group contained only medium without seeded cells. The cells were incubated with the CCK8 solution for 3 h, after which the optical density (OD) value of each well was measured at 450 nm using a microplate reader. The cell viability was calculated using Equation (2):

(2) $$\mathrm{Cell}\;\mathrm{viability}\;(\%)\;=\frac{\mathrm{OD}450(\mathrm{UVB})\;-\;\mathrm{OD}450(\mathrm{blank})}{\mathrm{OD}450(\mathrm{control})\;-\;\mathrm{OD}450(\mathrm{blank})}\;\times \;100$$

### 2.4. Determination of the ROS

To evaluate reactive oxygen species (ROS) levels, cells were initially seeded in 96-well plates. After a 24 h incubation period, reaching 50–60% confluence, the cells underwent UVB exposure in 50 μL PBS. Subsequently, they were treated with EU for an additional 24 h in a complete medium. ROS generation was assessed using fluorescent staining with 2′-7′dichlorofluorescin diacetate (DCFH-DA). After cellular uptake, the DCFH-DA was deacetylated using esterase to convert it into a non-fluorescent compound, which was subsequently oxidized by ROS to fluorescing 2′-7′dichlorofluorescein. The intracellular ROS levels were quantified using a fluorescence microplate reader (Thermo Varioskan Flash, Waltham, MA, USA) at excitation and emission wavelengths of 480 nm and 530 nm, respectively.

### 2.5. Animal Experiments

Previous studies have extensively used the SKH-1 hairless mouse strain as a skin photoaging model [21]. This paper also utilized SKH-1 hairless mice to investigate the anti-photoaging effects of EU in vivo according to the protocols established in previous research [22]. All experimental procedures were approved by the China Agricultural University Laboratory Animal Welfare and Animal Experimental Ethical Committee (Approval No: AW30803202-4-9, Beijing, China). Twenty-one female SKH-1 hairless mice, aged seven weeks, were obtained from Vital River Laboratories (Beijing, China) and housed at the Animal Center (Approval No: SYXK (Jing) 2020-0052) under specific pathogen-free conditions, which included a regulated environment with a 12 h light/dark cycle, a relative humidity between 40% and 70%, and a temperature of 22 ± 2 °C. After a one-week acclimation period, the mice were randomly assigned to three groups: the control, UVB, and EU+UVB groups (*n* = 7 per group). The dorsal skin of the mice in the UVB and EU+UVB groups received UVB irradiation three times per week. Three UVB lamps (TL 40W/12 RS SLV/25, Philips, Amsterdam, The Netherlands) were placed at a consistent distance of 30 cm from the dorsal skin to provide UVB irradiation. An irradiation intensity of 0.225 mW/cm^2^ was measured using a radiometer. The UVB doses were increased by 1 minimal erythemal dose (MED) in weekly increments, with 1 MED equivalent to 100 mJ/cm^2^. The dosage was gradually escalated until reaching a 3 MED level, which was maintained throughout the experiment. The UVB irradiation was terminated after 14 weeks. Both the control and UVB groups were fed an AIN93G diet, while the EU+UVB group received the same diet supplemented with 0.05% EU (*w*/*w*) (Table S1). The animals had unrestricted access to food and water throughout the study, while their food intake and body weights were regularly documented. Skin samples were collected after a 6 h morning fasting period from 8:00 a.m. to 2:00 p.m., rapidly frozen in liquid nitrogen, and stored at −80 °C for subsequent analysis.

### 2.6. Assessment of Wrinkle Formation

The mice were anesthetized prior to sacrifice, after which Silflo silicone was applied to the skin surfaces to create wrinkle replicas for wrinkle formation evaluation. The wrinkle severity was quantified using Primos CR (Canfield Scientific, Parsippany, NJ, USA), which measured various parameters, including average wrinkle depth, maximum wrinkle depth, and total wrinkle volume.

### 2.7. Determination of Skin Hydration

The dorsal skin was promptly excised after sacrifice, and the subcutaneous adipose tissue was carefully removed. About 0.2 g of skin was precisely weighed to determine the wet weight and immediately placed in an oven at 60 °C until reaching a constant weight. The skin hydration was calculated using Equation (3):

(3)  Skin hydration (%)=(wet weight − dry weight) / wet weight × 100

### 2.8. Analysis of the EU Accumulation in the Skin

The skin samples from the EU+UVB group (*n* = 3) were treated with 10 mL of acetonitrile through sonication, followed by centrifugation. The subsequent residues were subjected to additional extraction with 5 mL of acetonitrile, followed by sonication and centrifugation. The supernatants were pooled and degreased by adding 5 mL of acetonitrile-saturated n-hexane. The acetonitrile layer was retrieved after centrifugation and desiccated in a 45 °C water bath using nitrogen gas. The residues were reconstituted in 500 μL of ethyl acetate for gas chromatography-mass spectrometry (GC-MS). The prepared samples were injected into an Agilent 7890B GC-MS system (Agilent, Santa Clara, CA, USA), using a 2 × 15 m × 0.25 mm × 0.25 μm HP-5MS column for separation.

### 2.9. Determination of MDA, SOD, MMP-1, PIP, and HA Levels

The total protein was extracted from the cells and skin tissue using RIPA lysis buffer with proteinase inhibitors. The lipid peroxidation assay relied on the reaction between the MDA and thiobarbituric acid, forming an MDA-thiobarbituric acid adduct that exhibited strong absorbance at 532 nm. The SOD was assessed using the WST-8 method and a SOD assay kit, and absorbance was measured at 450 nm. The total cellular MMP-1, PIP, and HA content, as well as skin tissue HA levels, were estimated using the respective ELISA kits according to the protocols of the manufacturer. The absorbance was measured at 450 nm using a microplate reader. The final MDA, SOD, MMP-1, PIP, and HA levels were normalized to the total cellular or skin tissue protein content.

### 2.10. Hematoxylin and Eosin (HE) and Masson Staining

After the skin samples were fixed in formalin and embedded in paraffin, 5-μm sections were prepared, which were stained with HE and Masson’s trichrome manually. Microscopic examination was performed using an optical microscope (NIKON ECLIPSE E100, Nikon, Tokyo, Japan), and images were captured using a camera (NIKON DS-U3, Nikon, Tokyo, Japan) for further analysis. Image J software (version 1.53) was used to determine the epidermal thickness in HE-stained sections and the collagen density in the Masson-stained sections.

### 2.11. Immunohistochemistry Staining of p21 and p53

The paraffin blocks containing the skin samples were sectioned into 5 μm slices and heat-treated for 2 h at 65 °C. A citrate buffer (pH 6.0) was used for antigen retrieval at 121 °C for 2 min. After a blocking process with serum and hydrogen peroxide, the slices were incubated overnight at 4 °C with the anti-p21 (1:1000, Servicebio, Wuhan, China) and anti-p53 (1:200, Bioss, Beijing, China) primary antibodies. Next, the slices were incubated with a horseradish enzyme-labeled goat anti-rabbit immunoglobulin G antibody (1:200, Servicebio, Wuhan, China) at 37 °C for 30 min, stained with hematoxylin and 3,3′-diaminobenzidine. The stained slices were examined using an optical microscope (NIKON ECLIPSE E100, Nikon, Tokyo, Japan), and images were captured using a camera (NIKON DS-U3, Nikon, Tokyo, Japan). Finally, Image J software version 1.53 was employed to determine the positive area.

### 2.12. Real-Time Quantitative PCR (RT-qPCR)

The total RNA was extracted from the skin tissue using TRIzol. The RNA concentration was assessed using a Nano 300 spectrophotometer (Allsheng Co., Ltd., Hangzhou, China). TransScript One-Step gDNA Removal and cDNA Synthesis SuperMix (TransGen Biotech, Beijing, China) was used for cDNA synthesis, followed by RT-qPCR employing SuperReal PreMix Plus (SYBR Green) (TransGen Biotech, Beijing, China). Table S2 presents the primer sequences used for RT-qPCR. The mRNA expression levels were normalized to *β-actin* expression.

### 2.13. Skin Transcriptome

Total RNA was extracted from the skin samples of the control, UVB, and EU+UVB groups (*n* = 5 mice per group) using TRIzol^®^ reagent, while the genomic DNA was removed via DNase I treatment (Takara, Kyoto, Japan). An ND-2000 spectrophotometer (NanoDrop Technologies, Wilmington, DE, USA) was used to measure the RNA concentration, while the RNA purity was assessed via a 2100 Bioanalyzer (Agilent Technologies, Santa Clara, CA, USA). An Illumina TruSeq™ RNA sample preparation kit (San Diego, CA, USA) was employed to construct the RNA-Seq transcriptome library. An Illumina HiSeq X Ten/NovaSeq 6000 sequencer was used for paired-end RNA-Seq, producing reads with lengths of 2 × 150 bp, while TBS380 was used for library quantification. The raw paired-end read quality was assessed using SeqPrep (https://github.com/jstjohn/SeqPrep, accessed on 1 June 2023) and Sickle (https://github.com/najoshi/sickle, accessed on 1 June 2023), using default parameters for trimming and quality control. The clean reads, obtained after quality control, were aligned to the reference genome using HISAT2 (Version 2.1.0, http://ccb.jhu.edu/software/hisat2/index.shtml, accessed on 1 June 2023). A reference-based method employing the StringTie functionalities (Version 2.1.2, https://ccb.jhu.edu/software/stringtie/index.shtml?t=example, accessed on 1 June 2023) was used to construct mapped reads for each sample.

### 2.14. Differential Gene Expression and Functional Enrichment Analysis

The transcripts per million reads approach was applied to identify the differentially expressed genes (DEGs) and estimate the transcript expression levels. The DEGs were determined using the DESeq2 method, with a significance threshold of *p*-adjust < 0.05 and a |fold change| > 2. The Majorbio Cloud platform (www.majorbio.com, accessed on 15 July 2023) was used for the Kyoto Encyclopedia of Genes and Genomes (KEGG) analysis. The enrichment significance was determined at a *p*-adjust threshold of <0.05 to determine the top 20 KEGG pathways exhibiting the most substantial enrichment.

The GSEA software (Version 3.0) on the Majorbio Cloud platform (www.majorbio.com, accessed on 15 July 2023) was used for gene set enrichment analysis (GSEA), with default parameters of minGSSize = 15 and maxGSSize = 500. This analysis included all genes and phenotypes, aiming to identify the enriched gene sets associated with specific biological functions or pathways. The hallmark gene set from the MSigDB collections (version 6.2) was employed as the reference genome. The top 50 significantly altered pathways, based on the absolute value of the normalized enrichment score (*p*-value < 0.05), were selected from the GSEA results for further analysis.

The STRING database (Version 11.5, https://string-db.org, accessed on 1 August 2023) was employed to construct a protein–protein interaction (PPI) network using the queried genes, applying a minimum interaction score threshold of 0.700. The DEGs associated with the target pathways were entered into the STRING database, and the hub genes were identified as those with a node degree of seven or more.

Transcription factors (TFs) are proteins that control gene expression by binding to particular DNA sequences. Their primary function is controlling the transcription process, influencing target gene activity. ChEA3 is a web-based tool (Version 3, https://maayanlab.cloud/chea3, accessed on 1 August 2023) used for TF enrichment analysis, ranking the TFs associated with the submitted gene lists. It consolidates information from ENCODE, ReMap, and CHIP-Seq datasets, along with TF co-expression details from the RNA-Seq data sourced from the GTEx, TCGA, and ARCHS4 datasets. The top 10 TFs were identified after submitting the DEGs from the target pathways to ChEA3.

### 2.15. Fecal DNA Extraction and 16S rRNA Gene Sequencing

DNA from feces samples of the control, UVB, and EU+UVB groups (*n* = 6 mice per group) was extracted using a fecal DNA isolation kit (FUDEAN, Beijing, China). DNA concentration and purity were assessed using a NanoDrop 2000 spectrophotometer. The 16S rRNA gene segments (V3–V4) of the extracted DNA were amplified using the 806R (5′-GGACTACHVGGGTWTCTAAT-3′) and 338F (5′-ACTCCTACGGGAGGCAGCAG-3′) primers. The PCR conditions included 30 s at 95 °C, 30 s at 55 °C, and 45 s at 72 °C for 27 cycles. Then, the Illumina MiSeq sequencing platform and PE300 chemicals at Majorbio were used for the amplicon paired-end sequencing.

### 2.16. Microbiota Data Analysis

After demultiplexing, the generated sequences were merged using FLASH (version 1.2.11), after which fastp (version 0.19.6) was used for quality screening. The DADA2 plugin in the QIIME (version 2020.2) pipeline was employed to denoise the high-quality sequences and obtain the amplicon sequence variants (ASVs). The Naive Bayes consensus classifier in QIIME (version 2020.2) and the SILVA 16S rRNA database (version 138) were used to classify the ASVs. The α- and β-diversity was determined using QIIME, while the Majorbio Cloud platform was employed for principal coordinate analysis (PCoA) visualizations and unweighted_unifrac distance-based analysis of similarity. The differential bacterial taxa from phylum to genus were identified via linear discriminant analysis (LDA) effect size (LEfSe) using two filters (*p* < 0.05 and LDA score > 2). The ANCOM 4.0.2 package in R was employed for the Analysis of the Composition of Microbiomes (ANCOM) to enhance the identification of the enriched bacterial taxa in the control, UVB, and EU+UVB groups. The significance for W > 0.6 interpretations was defined. PICRUSt was used to predict the functional profiles of the microbial communities, while STAMP (Version 2.1.3, http://kiwi.cs.dal.ca/Software/STAMP, accessed on 1 September 2023) was employed to assess statistically significant differences and data analysis occurred on the Majorbio Cloud platform (www.majorbio.com, accessed on 1 September 2023). The Spearman’s correlation algorithm was used to establish the co-occurrence networks, where correlations with a *p*-value of <0.05 and a magnitude of ≥0.6 (indicating strong co-abundance relationships) or ≤−0.6 (indicating strong exclusion relationships) were deemed significant. Cytoscape V3.9.1 was used for co-occurrence visualization, while the default setting of the network analyzer algorithm in this program was employed to calculate the topological network parameters.

Spearman correlation analysis in SPSS version 17.0 (IBM, Armonk, NY, USA) was used for correlation analysis among skin photoaging-related indices, hub genes, and the relative gut microbiota abundance. The Spearman correlation analysis heatmap was created using the GraphPad Prism 9.4.0 software (San Diego, CA, USA).

### 2.17. Statistical Analysis

The data were presented as mean ± SEM. The statistical significance between the two groups was determined using a Student’s *t*-test. For comparisons between three groups, a one-way analysis of variance was applied to determine the significant differences. The statistical analyses were performed using the GraphPad Prism 9.4.0 software, with significance expressed as * *p* < 0.05, ** *p* < 0.01, *** *p* < 0.001, and **** *p* < 0.0001.

## 3. Results

### 3.1. EU Alleviates UVB-Induced Photoaging in HaCaT Epidermal Keratinocytes and Hs68 Dermal Fibroblasts

#### 3.1.1. EU Alleviates UVB-Induced Oxidative Damage in HaCaT Cells

**Figure 1:**
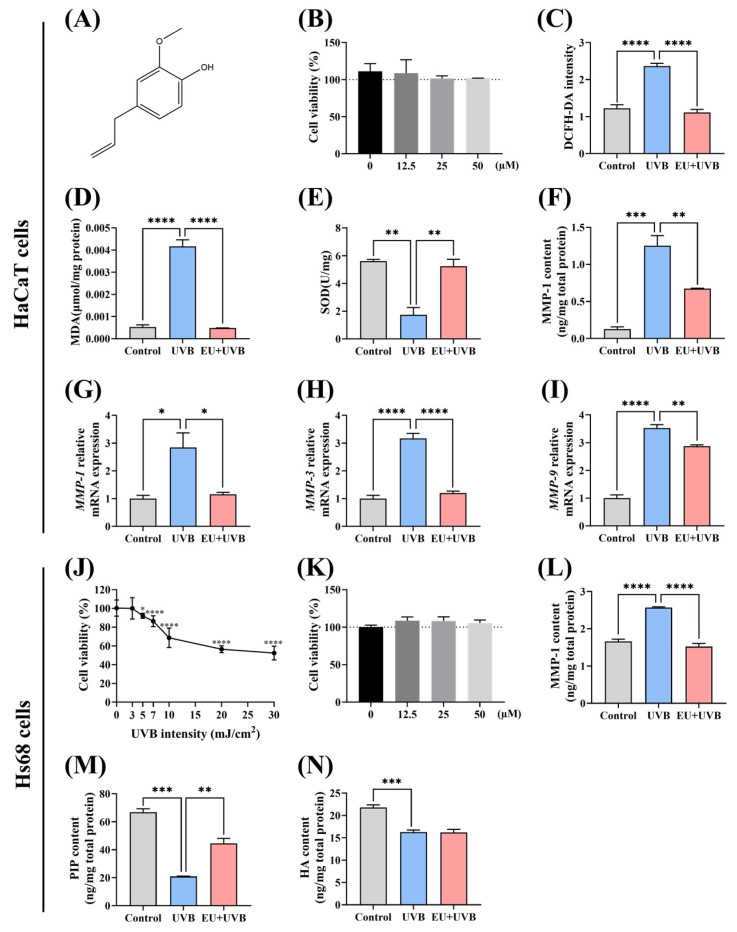
EU alleviates UVB-induced photoaging in HaCaT and Hs68 cells. (**A**) The chemical structure of EU. (**B**) Cell viability after 24 h incubation with 0, 12.5, 25, and 50 µM EU in HaCaT cells. *n* = 3. (**C**) ROS level in HaCaT cells. *n* = 8. (**D**) MDA content in HaCaT cells. *n* = 3. (**E**) SOD level in HaCaT cells. *n* = 3. (**F**) MMP-1 content in HaCaT cells. *n* = 3. (**G**) Relative mRNA expression of *MMP-1* in HaCaT cells. *n* = 3. (**H**) Relative mRNA expression of *MMP-3* in HaCaT cells. *n* = 3. (**I**) Relative mRNA expression of *MMP-9* in HaCaT cells. *n* = 3. (**J**) Cell viability of Hs68 cells irradiated with different UVB doses in Hs68 cells. *n* = 8. (**K**) Cell viability after 24 h incubation with 0, 12.5, 25, and 50 µM EU in Hs68 cells. *n* = 4. (**L**) MMP-1 content in Hs68 cells. *n* = 3. (**M**) PIP content in Hs68 cells. *n* = 3. (**N**) HA content in Hs68 cells. *n* = 3. Data were presented as mean ± SEM. Significant differences between groups are indicated as: * *p* < 0.05; ** *p* < 0.01; *** *p* < 0.001; **** *p* < 0.0001.

A CCK8 assay was used to explore the impact of EU (Figure 1A) on HaCaT cell viability. An EU concentration of 50 µmol/L was selected for the subsequent experiments since the results indicated that EU treatment at this level exhibited no significant cytotoxicity in the HaCaT cells (Figure 1B). A UVB irradiation dose of 5 mJ/cm^2^ was applied to induce photoaging in the HaCaT cells [23]. The ROS, MDA, and SOD levels were evaluated to determine the efficacy of EU in mitigating UVB-induced oxidative stress in HaCaT cells. UVB radiation significantly increased the ROS levels and lipid oxidation MDA content in the HaCaT cells (Figure 1C,D), while the intracellular antioxidant enzyme SOD activity displayed a substantial decline (Figure 1E). EU treatment effectively suppressed the UVB-induced rise in intracellular ROS levels (Figure 1C), efficiently decreased MDA accumulation (Figure 1D), and successfully restored the intracellular SOD levels to the normal state (Figure 1E). These results demonstrated the efficacy of EU treatment in reducing the oxidative damage caused by UVB exposure in HaCaT cells. In addition, EU treatment had no significant effect on the levels of MDA and SOD in normal HaCaT (Figure S1A,B).

#### 3.1.2. EU Inhibits the UVB-Induced Increase in MMP Secretion in HaCaT Cells

UVB irradiation notably increased MMP-1 protein secretion (Figure 1F). EU treatment restored the MMP-1 expression in the UVB-induced HaCaT cells (Figure 1F). Furthermore, assessment according to the ELISA results showed that UVB radiation substantially increased *MMP-1*, *MMP-3*, and *MMP-9* mRNA expression levels in the HaCaT cells, while EU treatment decreased these levels (Figure 1G–I). These findings demonstrated the efficacy of EU treatment in suppressing the UVB-induced MMP expression elevation in HaCaT cells. EU intervention appeared to have no impact on the levels of MMP-1 protein, as well as *MMP-1*, *MMP-3*, and *MMP-9* genes in normal HaCaT cells (Figure S1C–F).

#### 3.1.3. EU Attenuates the UVB-Induced Decrease of ECM in Hs68 Cells

The optimal UVB irradiation dose was determined by exposing the Hs68 cells to different UVB radiation levels (0 mJ/cm^2^, 3 mJ/cm^2^, 5 mJ/cm^2^, 7 mJ/cm^2^, 10 mJ/cm^2^, 20 mJ/cm^2^, and 30 mJ/cm^2^), after which the cell viability was evaluated using a CCK8 kit. Compared with unexposed Hs68 cells, UVB irradiation in a range of 3 mJ/cm^2^ to 30 mJ/cm^2^ dose-dependently reduced cell proliferation (Figure 1J). Therefore, a UVB dose of 5 mJ/cm^2^ was selected for Hs68 cell irradiation. An EU concentration of 50 µmol/L was selected for the subsequent experiments since treatment at this level presented no notable cytotoxicity in the Hs68 cells (Figure 1K). UVB exposure promoted MMP-1 protein secretion and reduced that of collagen (represented by the PIP content) and HA in the Hs68 cells (Figure 1L–N). However, although EU treatment suppressed the UVB-induced increase in MMP-1 secretion and restored the collagen content (Figure 1L,M), it did not appear to remediate the HA content (Figure 1N). In conclusion, EU attenuated the UVB-induced decrease of ECM in the Hs68 cells. EU treatment did not appear to have a significant effect on the contents of MMP-1 protein and collagen in normal Hs68 cells (Figure S1G,H).

### 3.2. Dietary EU Mitigates the Wrinkle Formation Induced by Chronic UVB Exposure in the Skin of SKH-1 Hairless Mice

**Figure 2:**
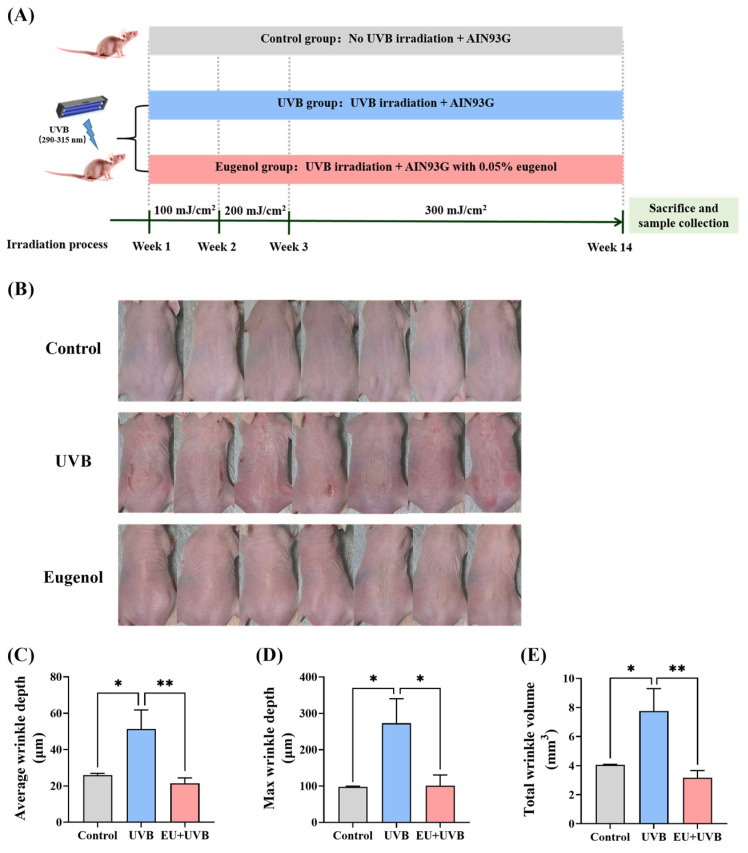
EU suppresses wrinkle formation in chronic UVB-exposed mice. (**A**) Schematic representation of the experimental procedure. (**B**) Images of mouse dorsal skin at week 14 of the experiment. *n* = 7. (**C**) Average wrinkle depth. *n* = 6. (**D**) Max wrinkle depth. *n* = 6. (**E**) Total wrinkle volume. *n* = 6. Data were presented as mean ± SEM. Significant differences between groups are indicated as: * *p* < 0.05; ** *p* < 0.01.

Figure 2A delineates the detailed experimental schedule. In the present study, neither UVB exposure nor dietary EU affected the body weight and food intake of the mice during the experiment compared with the control group (Figure S2). Compared with the unirradiated mice, the dorsal skin of those exposed to chronic UVB exposure exhibited wrinkle formation, which was significantly reduced by dietary EU (Figure 2B). The wrinkle depth and volume were assessed using silicone replicas. The results confirmed that chronic UVB exposure promoted wrinkle formation, characterized by an increase in the average wrinkle depth, max wrinkle depth, and total wrinkle volume, while EU supplementation significantly reduced these parameters (Figure 2C–E), highlighting its efficacy in alleviating the adverse effect of chronic UVB exposure on the development of wrinkles.

### 3.3. The EU Distribution in the Skin of Chronically UVB-Exposed Mice

After the 14-week intervention, the skin concentrations of the EU+UVB group mice were measured using GC-MS to examine the effect of EU on skin photoaging. The EU content in the skin was determined as 0.06 ± 0.02 μmol/kg (Table S3).

### 3.4. EU Promotes Skin Barrier Repair in Chronically UVB-Exposed Mice

**Figure 3:**
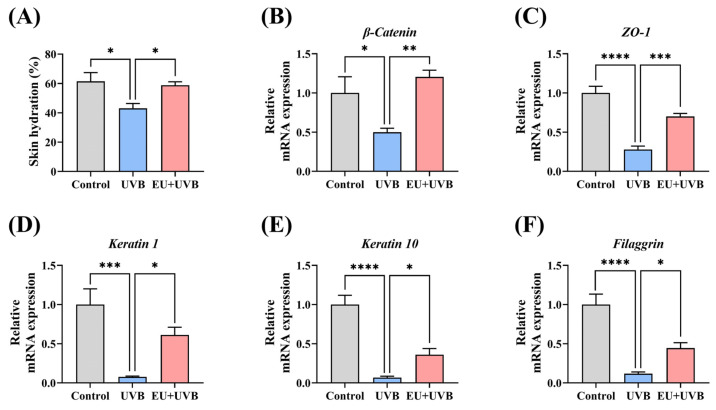
EU promotes skin barrier repair in chronic UVB-exposed mice. (**A**) Skin hydration. *n* = 7. (**B**) Relative mRNA expression of *β-catenin*. *n* = 5. (**C**) Relative mRNA expression of *ZO-1*. *n* = 5. (**D**) Relative mRNA expression of *keratin 1*. *n* = 5. (**E**) Relative mRNA expression of *keratin 10*. *n* = 5. (**F**) Relative mRNA expression of *filaggrin*. *n* = 5. Data were presented as mean ± SEM. Significant differences between groups are indicated as: * *p* < 0.05; ** *p* < 0.01; *** *p* < 0.001; **** *p* < 0.0001.

The impact of EU on skin barrier restoration in photoaged mice was assessed by examining skin hydration and barrier protein-related gene expression. The results indicated that dietary EU restored skin hydration caused by prolonged UVB irradiation, consequently alleviating skin dryness (Figure 3A). Furthermore, the gene expression of the crucial barrier proteins in the skin was investigated. The findings revealed a significant reduction in the *β-catenin* adhesion linker protein, the *ZO-1* tight junction protein, the *keratin 1* and *keratin 10* keratins, and the aggregating protein *filaggrin* aggregating protein levels in the skin of mice exposed to prolonged UVB irradiation, while EU treatment significantly restored the gene expression levels of these barrier proteins (Figure 3B–F). Therefore, these findings suggest that EU promotes barrier repair in photoaged mice.

### 3.5. EU Promotes Skin Tissue Regeneration in Chronically UVB-Exposed Mice

#### 3.5.1. EU Mitigates the Chronic UVB-Induced Increase in Epidermal Thickness in Mice

**Figure 4:**
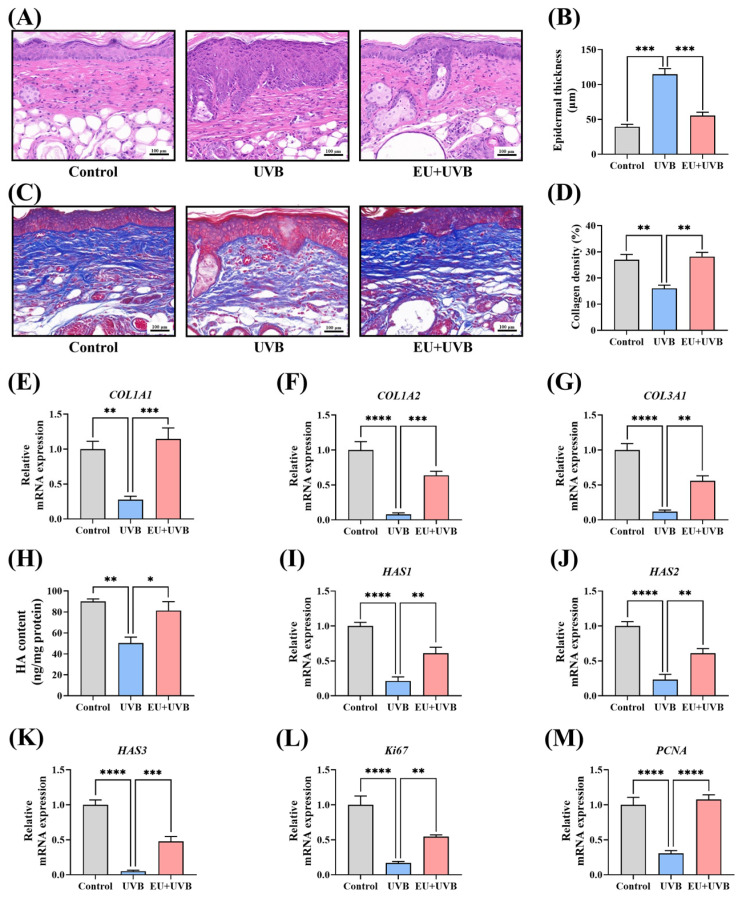
EU boosts skin tissue regeneration in chronic UVB-exposed mice. (**A**) Representative micrographs of HE-stained skin tissue sections after UVB irradiation for 14 weeks (100×, Scale bar = 100 µm). (**B**) Average epidermal thickness of the dorsal skin. *n* = 3. (**C**) Representative micrographs of Masson-stained skin tissue sections after UVB irradiation for 14 weeks (100×, Scale bar = 100 µm). (**D**) Average collagen density of the dorsal skin. *n* = 3. (**E**) Relative mRNA expression of *COL1A1*. *n* = 5. (**F**) Relative mRNA expression of *COL1A2*. *n* = 5. (**G**) Relative mRNA expression of *COL3A1*. *n* = 5. (**H**) HA contents. *n* = 3. (**I**) Relative mRNA expression of *HAS1*. *n* = 5. (**J**) Relative mRNA expression of *HAS2*. *n* = 5. (**K**) Relative mRNA expression of *HAS3*. *n* = 5. (**L**) Relative mRNA expression of *ki67*. *n* = 5. (**M**) Relative mRNA expression of *PCNA*. *n* = 5. Data were presented as mean ± SEM. Significant differences between groups are indicated as: * *p* < 0.05; ** *p* < 0.01; *** *p* < 0.001; **** *p* < 0.0001.

The skin samples were stained with HE for histological analysis, revealing substantial epidermal thickening in the mice exposed to chronic UVB irradiation, which was notably reduced by dietary EU supplementation (Figure 4A,B).

#### 3.5.2. EU Promotes ECM Synthesis in Photodamaged Mouse Skin

The mouse skin tissue was subjected to Masson staining to investigate the impact of EU on the collagen fiber content. Continuous exposure to UVB decreased the collagen fiber density in the dorsal skin of the mice, which was restored by dietary EU supplementation (Figure 4C,D). Moreover, compared to unirradiated mice, the relative mRNA expression levels of the collagen type I alpha 1 chain (*COL1A1*), collagen type I alpha 2 chain (*COL1A2*), and collagen type III alpha 1 chain (*COL3A1*) decreased significantly in the photoaged mice, which were substantially increased by dietary EU supplementation (Figure 4E–G). Additionally, the HA content, another essential component of the skin ECM, was evaluated. Chronic UVB exposure significantly decreased the HA level in the skin, while EU supplementation effectively restored the HA levels in the photoaged mouse skin (Figure 4H). The expression of HA synthase genes, including *HAS1*, *HAS2*, and *HAS3*, was significantly reduced in the skin of the photoaged mice, which was substantially upregulated by EU supplementation (Figure 4I–K). Therefore, the findings indicate that EU supplementation promotes ECM synthesis in the skin of photoaged mice.

#### 3.5.3. EU Promotes Skin Cell Proliferation in Photoaged Mice

The expression of the *ki67* and *PCNA* proliferation markers was analyzed to determine whether EU promoted skin cell proliferation in photodamaged mice. The results showed that chronic UVB exposure significantly decreased the *ki67* and *PCNA* levels in the mouse skin, which were effectively restored by EU intervention (Figure 4L,M). This suggests that dietary EU promotes skin cell proliferation.

### 3.6. EU Modulates the Skin Microenvironment in Chronically UVB-Exposed Mice

#### 3.6.1. EU Alleviates Oxidative Stress in Photoaged Mouse Skin

**Figure 5:**
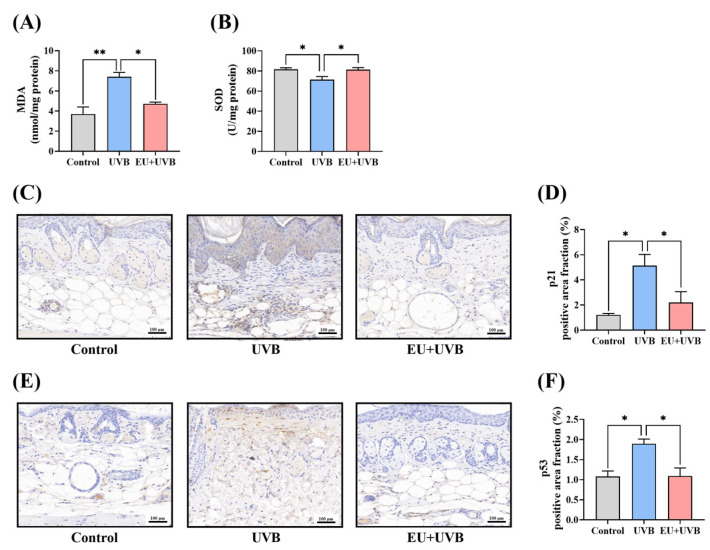
EU modulates the skin microenvironment in chronic UVB-exposed mice. (**A**) MDA contents. *n* = 3. (**B**) SOD activity. *n* = 3. (**C**) Representative micrographs of p21 immunohistochemically stained skin tissue sections after UVB irradiation for 14 weeks (200×, Scale bar = 100 µm). (**D**) Average p21 positive area fraction in the dorsal skin. *n* = 3. (**E**) Representative micrographs of p53 immunohistochemically stained skin tissue sections after UVB irradiation for 14 weeks (200×, Scale bar = 100 µm). (**F**) Average p53 positive area fraction in the dorsal skin. *n* = 3. Data were presented as mean ± SEM. Significant differences between groups are indicated as: * *p* < 0.05; ** *p* < 0.01.

Oxidative stress damage is an important manifestation of skin photoaging. UVB exposure significantly increased the MDA content and substantially reduced the SOD activity (Figure 5A,B). EU supplementation mitigated this oxidative stress damage by lowering the MDA level and restoring the SOD activity (Figure 5A,B).

#### 3.6.2. EU Reduces the Expression of the p21 Senescence Marker and p53 DNA Damage Marker in the Skin of Mice Exposed to Chronic UVB Irradiation

The impact of dietary EU supplementation on the expression of the p21 aging marker in the mouse skin was investigated. Compared with the unirradiated mice, UVB exposure significantly increased the p21 level in the dorsal skin of the mice, which was reduced by dietary EU intervention (Figure 5C,D). Additionally, chronic UVB exposure induced DNA damage in the mouse skin, increasing the p53 DNA damage marker level, which was significantly decreased by dietary EU supplementation (Figure 5E,F).

### 3.7. EU Modifies the Gene Expression Profiles in the Skin

**Figure 6:**
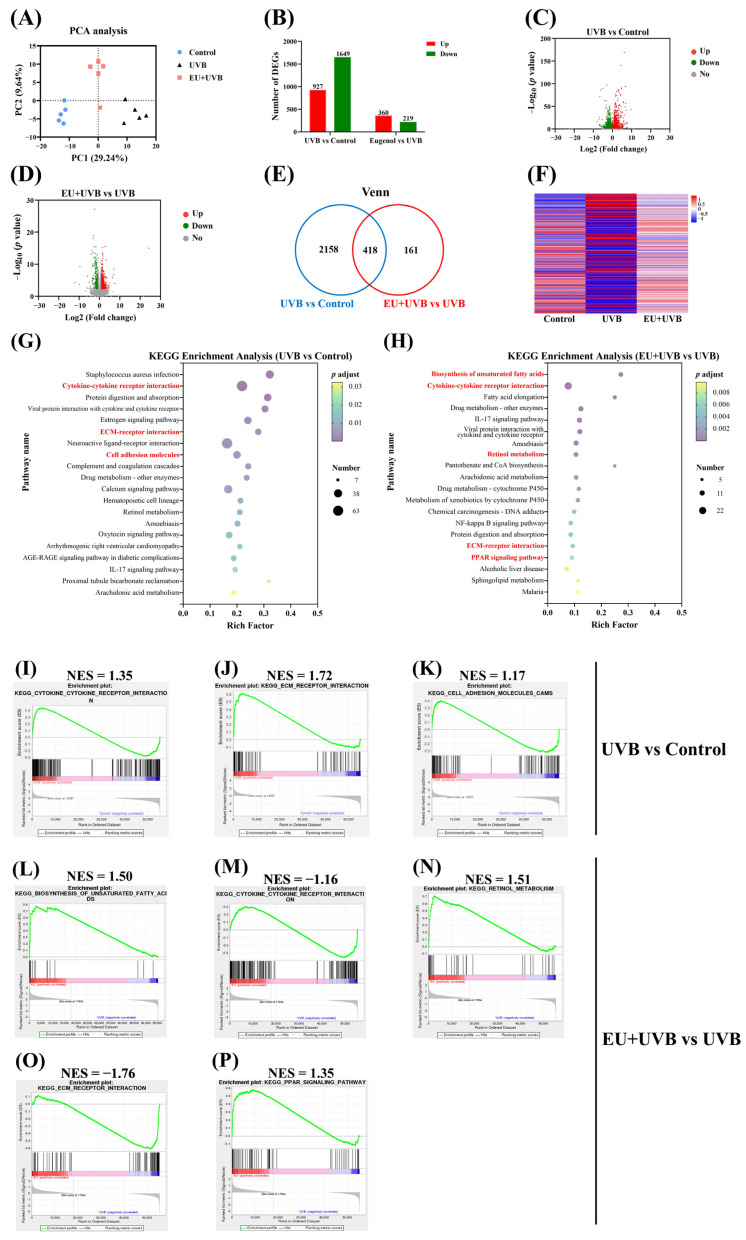
EU modifies gene expression profiles and reverses ECM–receptor interaction and cytokine–cytokine receptor interaction in the skin of chronic UVB-exposed mice. (**A**) PCA of the skin transcriptome. (**B**) Numbers of DEGs in the control, UVB, and EU+UVB groups. (**C**) Volcano plot of the DEGs in the UVB group mice compared with the control group mice. (**D**) Volcano plot of the DEGs in the EU+UVB group mice compared with the UVB group mice. (**E**) Venn analysis showed that 418 genes were overlapped based on the DEGs that are regulated in UVB vs. control and EU vs. UVB. (**F**) The heatmap of 418 overlapped DEGs. (**G**) KEGG enrichment analysis of DEGs between the UVB and control groups (*p* adjust < 0.05). (**H**) KEGG enrichment analysis of DEGs between the EU and UVB groups (*p* adjust < 0.05). (**I**) Cytokine–cytokine receptor interaction enriched by GESA analysis in the UVB group compared to the control group. (**J**) ECM–receptor interaction enriched by GESA analysis in the UVB group compared to the control group. (**K**) Cell adhesion molecules enriched by GESA analysis in the UVB group compared to the control group. (**L**) Biosynthesis of unsaturated fatty acids enriched by GESA analysis in the EU+UVB group compared to the UVB group. (**M**) Cytokine–cytokine receptor interaction enriched by GESA analysis in the EU+UVB group compared to the UVB group. (**N**) Retinol metabolism enriched by GESA analysis in the EU+UVB group compared to the UVB group. (**O**) ECM–receptor interaction enriched by GESA analysis in the EU+UVB group compared to the UVB group. (**P**) PPAR signaling pathway enriched by GESA analysis in the EU+UVB group compared to the UVB group. NES, normalized enrichment score. *n* = 5.

To further elucidate the mechanism by which EU ameliorated skin photoaging, its influence on the transcriptome characteristics was investigated via RNA sequencing analysis of the dorsal skin of mice in the control, UVB, and EU+UVB groups. PCA indicated distinct differences between the skin transcriptomes of the groups (Figure 6A), revealing 2576 DEGs, of which 972 were upregulated, and 1649 were downregulated in the UVB group compared to the control group (Figure 6B,C). Furthermore, 579 DEGs were identified in the EU+UVB group, of which 360 were upregulated, and 219 were downregulated (Figure 6B,D). Venn analysis highlighted 418 genes displaying significant regulatory changes in both the UVB vs. control and EU vs. UVB groups (Figure 6E). The heatmap depicting these 418 overlapped DEGs suggests that EU reverses the effect of chronic UVB exposure, leading to a gene expression pattern more closely resembling that of the control group (Figure 6F).

### 3.8. EU Reverses Cytokine–Cytokine Receptor Interaction and ECM–Receptor Interaction in the Skin of Chronically UVB-Exposed Mice

This study employed two complementary approaches to gain a more comprehensive understanding of gene functions and crucial molecular pathways. KEGG enrichment analysis used the DEG lists as input, while GSEA analyzed the complete list of expressed genes. GSEA is a powerful analytical approach used to decipher gene expression data by assessing gene functions at the gene set level [24]. KEGG enrichment analysis of the 2576 DEGs between the UVB and control groups revealed that chronic UVB exposure influenced several pathways in the mouse skin, including cytokine–cytokine receptor interaction, ECM–receptor interaction, cell adhesion molecules, and the IL-17 signaling pathway (Figure 6G). KEGG enrichment analysis of the 579 DEGs between the EU and UVB groups revealed that prolonged UVB irradiation affected numerous pathways in the exposed mice, including unsaturated fatty acid biosynthesis, cytokine–cytokine receptor interaction, retinol metabolism, ECM–receptor interaction, and the PPAR signaling pathway (Figure 6H). Table S4 presents the GSEA enrichment analysis results after utilizing all the genes in the control and UVB groups (top 50, *p* < 0.05). Table S5 presents the GSEA enrichment analysis results after using all the genes in the UVB and EU+UVB groups (top 50, *p* < 0.05). The KEGG and GSEA analyses showed cytokine–cytokine receptor interaction, ECM–receptor interaction, and cell adhesion molecule overlapping between the control and UVB groups, representing the main pathways affected by chronic UVB exposure (Figure 6I–K). Additionally, the KEGG and GSEA analyses indicated unsaturated fatty acid biosynthesis, cytokine–cytokine receptor interaction, retinol metabolism, ECM–receptor interaction, and PPAR signaling pathway overlapping between the EU and UVB groups, confirming their status as major pathways affected by EU intervention (Figure 6L–P). Therefore, the results showed that dietary EU supplementation reversed the upregulation of the cytokine–cytokine receptor interaction and ECM–receptor interaction caused by chronic UVB exposure. It is hypothesized that the ability of EU to alleviate skin photoaging in mice may be associated with its capacity to regulate cytokine–cytokine receptor interaction and ECM–receptor interaction, consequently representing the key pathways influenced by EU intervention.

### 3.9. PPI and TF Target Enrichment Analysis of the DEGs in the Key Pathways Influenced by EU Intervention

**Figure 7:**
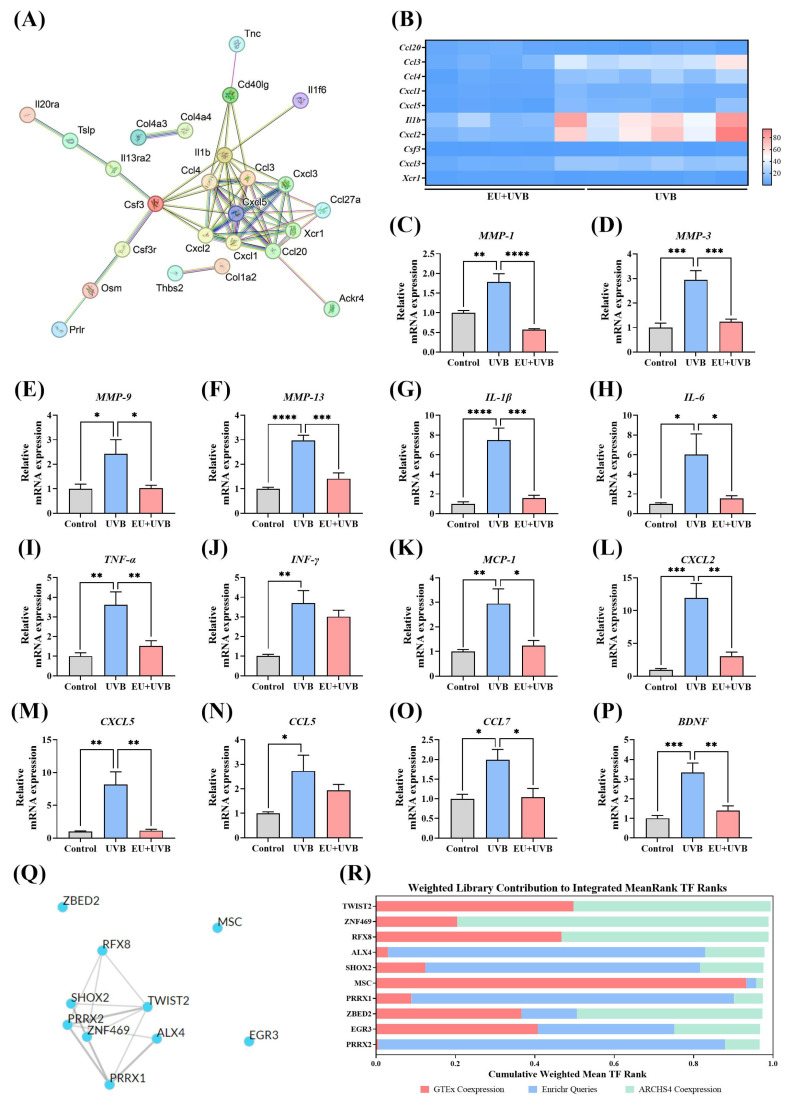
PPI and TF target enrichment analysis of DEGs on key pathways influenced by EU intervention. (**A**) STRING network visualization of the DEGs on ECM–receptor interaction and cytokine–cytokine receptor interaction. Edges represent protein–protein associations and the line thickness indicates the strength of data support. Genes associated with 7 or more proteins are identified as hub genes. (**B**) Heatmap of hub genes analyzed by RNA-seq. (**C**) Relative mRNA expression of *MMP-1*. (**D**) Relative mRNA expression of *MMP-3*. (**E**) Relative mRNA expression of *MMP-9*. (**F**) Relative mRNA expression of *MMP-13*. (**G**) Relative mRNA expression of *IL-1β*. (**H**) Relative mRNA expression of *IL-6*. (**I**) Relative mRNA expression of *TNF-α*. (**J**) Relative mRNA expression of *INF-γ*. (**K**) Relative mRNA expression of *MCP-1*. (**L**) Relative mRNA expression of *CXCL2*. (**M**) Relative mRNA expression of *CXCL5*. (**N**) Relative mRNA expression of *CCL5*. (**O**) Relative mRNA expression of *CCL7*. (**P**) Relative mRNA expression of *BDNF*. (**Q**) Top 10 TFs network generated by ChEA3 using the average integrated ranks across all libraries. (**R**) Top 10 TFs generated by ChEA3 based on Mean Rank ranking. For (**C**–**P**), data were presented as mean ± SEM, and significant differences between groups are indicated as: * *p* < 0.05; ** *p* < 0.01; *** *p* < 0.001; **** *p* < 0.0001. *n* = 5.

The STRING 10 database was used for PPI network analysis to determine the crucial gene targets affected by EU in the key pathways and identify genes with a node degree equal to or greater than seven as hub genes. The results showed that EU regulated 10 hub genes, namely *ccl20*, *ccl3*, *ccl4*, *cxcl1*, *cxcl5*, *IL-1β*, *cxcl2*, *csf3*, *cxcl3*, and *xcr1* (Figure 7A). The RNA-Seq results revealed that *ccl3*, *ccl4*, *cxcl1*, *cxcl5*, *IL-1β*, *cxcl2*, *csf3*, and *cxcl3* were downregulated, while *ccl20* and *xcr1* were upregulated in the mouse skin of the EU+UVB group compared to the UVB group (Figure 7B). Since these hub genes were mostly SASPs, RT-qPCR was used to analyze the alterations after EU intervention regarding the presence of *MMP-1*, *MMP-3*, *MMP-9*, *MMP-13*, interleukin (*IL*)-1β, *IL-6*, tumor necrosis factor-α (*TNF-α*), interferon-γ (*INF-γ*), monocyte chemoattractant protein-1 (*MCP-1*), C-X-C motif chemokine (*CXCL*) 2, *CXCL5*, C-C motif ligand (*CCL*) 5, *CCL7*, and brain-derived neurotrophic factor (*BDNF*). Compared to the unirradiated mice, the relative mRNA expression levels of *MMP-1*, *MMP-3*, *MMP-9*, *MMP-13*, *IL-1β*, *IL-6*, *TNF-α*, *INF-γ*, *MCP-1*, *CXCL2*, *CXCL5*, *CCL5*, *CCL7*, and *BDNF* increased significantly in the skin of the chronically UVB exposed group, while EU intervention effectively inhibited the expression of these SASPs (Figure 7C–P). These findings further confirmed the precision of the transcriptome sequencing outcomes. A TF enrichment analysis of the DEGs in the key pathways was performed using ChEA3 to explore the potential upstream TFs associated with the observed variations. Mean Rank ranking identified the top 10 TFs as TWIST2, ZNF469, RFX8, ALX4, SHOX2, MSC, PRRX1, ZBED2, EGR3, and PRRX2 (Figure 7Q,R).

### 3.10. EU Modulates the Diversity and Composition of Gut Microbiota in Photoaged Mice

**Figure 8:**
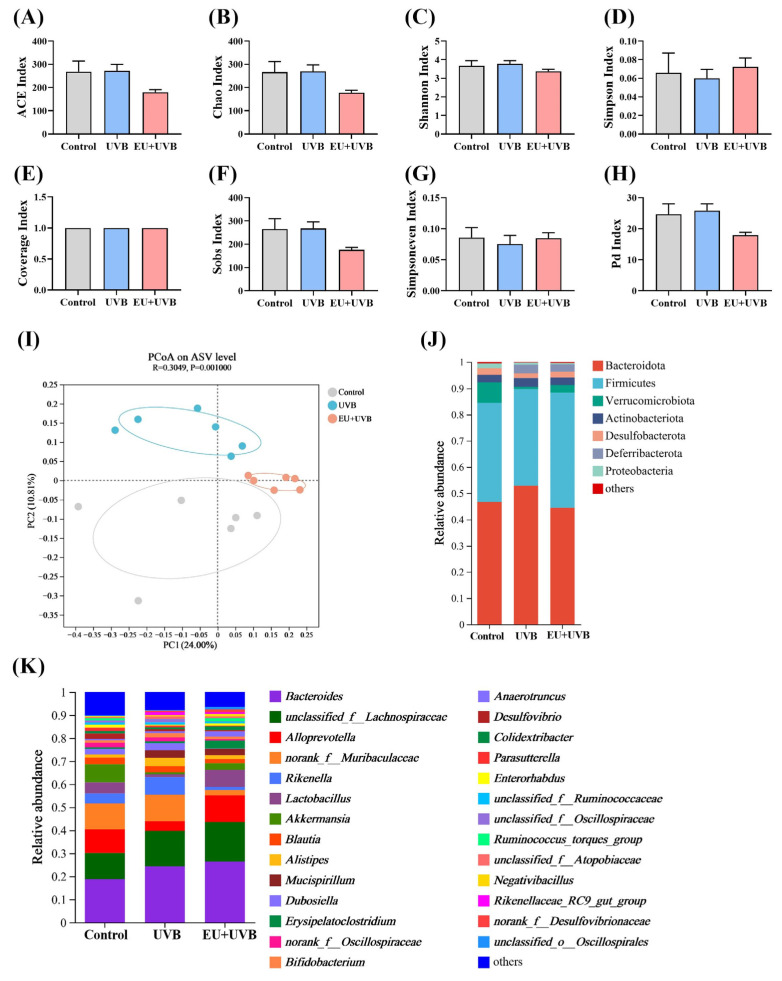
EU modulates the diversity and composition of gut microbiota in photoaged mice. (**A**) ACE index. (**B**) Chao index. (**C**) Shannon index. (**D**) Simpson index. (**E**) Coverage index. (**F**) Sobs index. (**G**) Simpsoneven index. (**H**) Pd index. (**I**) PCoA on ASV level based on unweighted_unifrac distance and assessed by analysis of similarity. (**J**) The relative abundance of gut microbiota at the phylum level. (**K**) The relative abundance of gut microbiota at the genus level. For (**A**–**H**), data were presented as mean ± SEM. *n* = 6.

DNA was extracted from the fecal samples of the mice in the control, UVB, and EU+UVB groups and subjected to 16S rRNA sequencing to investigate the regulatory effect of EU intervention on the gut microbiota. The impact of EU supplementation on the microbial communities was assessed via ASV analysis. Chronic UVB exposure and EU intervention did not appear to affect α-diversity measures, including the ACE index, Chao index, Shannon index, Simpson index, Coverage index, Sobs index, Simpsoneven index, and Pd index (Figure 8A–H). In terms of β-diversity, PCoA conducted at the ASV level using unweighted_unifrac distances displayed a clear separation between the gut microbiota structures of the control, UVB, and EU+UVB groups (Figure 8I), suggesting significant modifications due to chronic UVB exposure and EU supplementation. The altered gut microbial community structures in the control, UVB, and EU+UVB groups were linked to distinct changes in the relative abundance patterns, evident at both the phylum and genus levels (Figure 8J,K).

### 3.11. Characteristic Bacterial, Functional Prediction, and Co-Occurrence Network Analyses of the Gut Microbiota in the Photoaged Mice after EU Intervention

**Figure 9:**
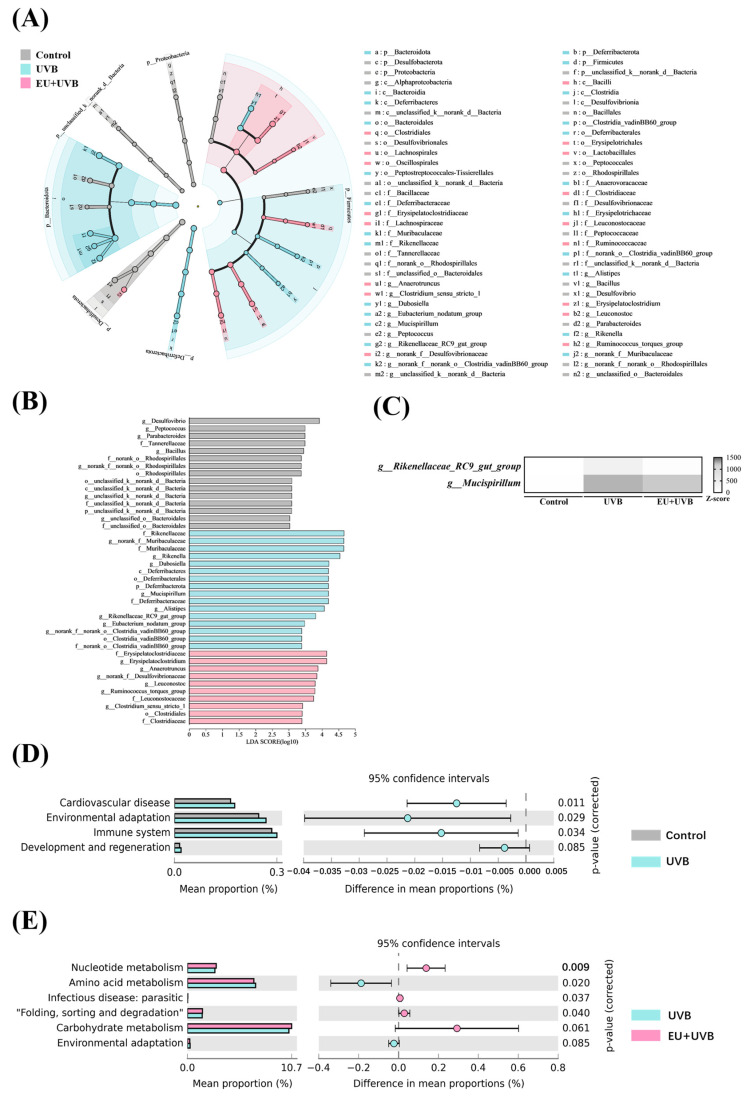
Characteristic bacteria analysis and functional prediction analysis of gut microbiota in photoaged mice after EU intervention. (**A**) LEfSe analysis showed the relationship of ASVs (the rings, from outer to inner, represent genus, family, order, class, and phylum). (**B**) Comparison of gut microbiota among the control, UVB, and EU+UVB groups with LDA score > 2. (**C**) A heatmap depicts the differential abundance of characteristic microbial taxa. Rows (microbial taxa at the genus level) and columns (samples) were ordered by hierarchical clustering. Row-wise Z-score scaling was conducted in the heatmap visualization, showing the normalized relative abundance by the mean and standard deviation of the specific taxa across all samples. (**D**) Predicted function of the fecal microbiome based on KEGG pathways between the control and UVB groups. (**E**) Predicted function of the fecal microbiome based on KEGG pathways between the UVB and EU+UVB groups. Statistical significance difference was performed using *t*-test (*p* < 0.05) in STAMP. *n* = 6.

LEfSe analyses were conducted at the ASV level with a scoring threshold of 2 to identify the differentially abundant bacterial taxa in the control, UVB, and EU+UVB groups of mice. The differentially abundant bacterial taxa in the gut microbiota of the control group mice were *g__Desulfovibrio*, *g__Peptococcus*, *g__Parabacteroides*, *f__Tannerellaceae, g__Bacillus*, *f__norank_o__Rhodospirillales*, *g__norank_f__norank_o__Rhodospirillales*, *o__Rhodospirillales*, *o__unclassified_k__norank_d__Bacteria*, *c__unclassified_k__norank_d__Bacteria*, *g__unclassified_k__norank_d__Bacteria*, *f__unclassified_k__norank_d__Bacteria*, *p__unclassified_k__norank_d__Bacteria*, *g__unclassified_o__Bacteroidales*, and *f__unclassified_o__Bacteroidales* (Figure 9A,B). The differentially abundant bacterial taxa in the gut microbiota of the UVB group mice were *f__Rikenellaceae*, *g__norank_f__Muribaculaceae*, *f__Muribaculaceae*, *g__Rikenella*, *g__Dubosiella*, *c__Deferribacteres*, *o__Deferribacterales*, *p__Deferribacterota*, *g__Mucispirillum*, *f__Deferribacteraceae*, *g__Alistipes*, *g__Rikenellaceae_RC9_gut_group*, *g__Eubacterium_nodatum_group*, *g__norank_f__norank_o__Clostridia_vadinBB60_group*, *o__Clostridia_vadinBB60_group*, and *f__norank_o__Clostridia_vadinBB60_group* (Figure 9A,B). The differentially abundant bacterial taxa in the gut microbiota of the EU+UVB group mice were *f__Erysipelatoclostridiaceae*, *g__Erysipelatoclostridium*, *g__Anaerotruncus*, *g__norank_f__Desulfovibrionaceae*, *g__Leuconostoc*, *g__Ruminococcus_torques_group*, *f__Leuconostocaceae*, *g__Clostridium_sensu_stricto_1*, and *o__Clostridiales,* and *f__Clostridiaceae* (Figure 9A,B). Considering the potential for higher false-positive rates when using LEfSe, the ANCOM method was employed to enhance the identification accuracy of the enriched bacterial taxa across group comparisons [25]. At a W > 0.6 significance level, *g__norank_f__Desulfovibrionaceae*, *g__Mucispirillum*, and *g__Rikenellaceae_RC9_gut_group* were differentially abundant among the groups at the genus level (Table S6), which were also identified in the LEfSe analysis. EU intervention alleviated the elevated abundance of *Rikenellaceae_RC9_gut_group* and *Mucispirillum* in the photoaged mice, while that of *norank_f__Desulfovibrionaceae* continued to increase after EU supplementation (Table S7). The combined results of the LEfSe analysis, ANCOM analysis, and the abundance change after EU supplementation identified *Rikenellaceae_RC9_gut_group* and *Mucispirillum* as the characteristic gut bacteria affected by EU (Figure 9C).

The 16S rRNA gene catalogs were annotated against the KEGG databases to delineate the microbial community functionality alterations after chronic UVB exposure and EU dietary supplementation. Four pathways were enriched after chronic exposure to UVB, namely cardiovascular disease, environmental adaptation, immune system, and development and regeneration (Figure 9D), while EU intervention upregulated nucleotide metabolism, infectious disease: parasitic, folding, sorting, and degradation, and carbohydrate metabolism, and downregulated amino acid metabolism and environmental adaptation (Figure 9E).

**Figure 10:**
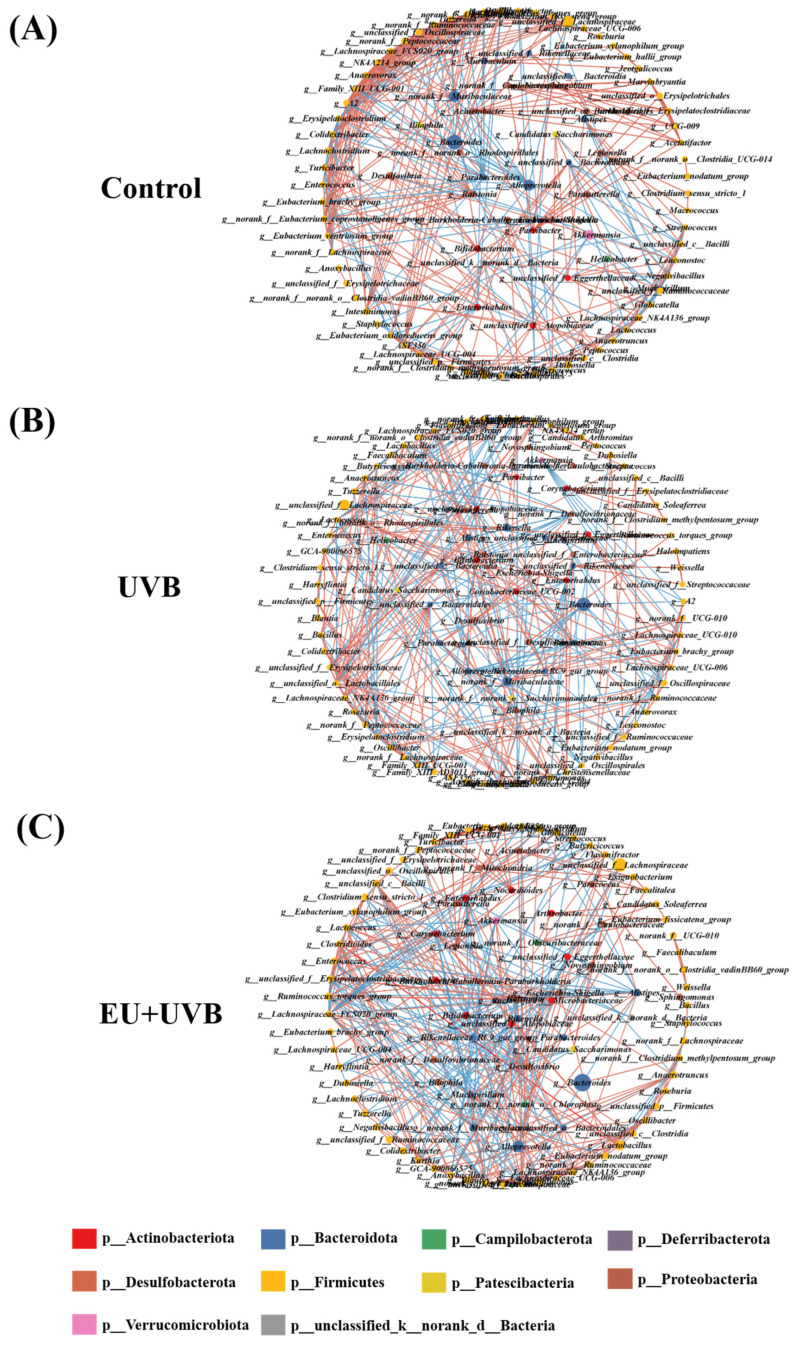
Co-occurrence network analysis of gut microbiota in photoaged mice after EU intervention. (**A**) Co-occurrence network of the control group. (**B**) Co-occurrence network of the UVB group. (**C**) Co-occurrence network of the EU+UVB group. The edges indicate statistically significant (*p* < 0.05) Spearman correlations (|r| > 0.6) between genera. Blue lines represent a significant negative correlation, and red lines represent a significant positive correlation. The size of the points represents the degree of the node, which are colored based on their affiliated phyla. *n* = 6.

Co-occurrence networks were constructed that considered the significant correlations (*p* < 0.05, |r| > 0.6) to clarify the relationships among the bacterial genera in the microbiome of photoaged mice supplemented with EU. The results revealed complex gut microbiota co-occurrence networks in the three groups of mice (Figure 10A–C). The co-occurrence network of the control group consisted of 98 nodes and 403 edges, while that of the UVB group presented 105 nodes and 383 edges, and the EU+UVB group included 100 nodes and 381 edges (Table S8). Further analysis of the co-occurrence networks showed that the path length, average degree, and graph density became more akin to those in the control group after EU intervention. This indicated that mice treated with EU tended to align their gut microbiota feature network with that of healthy mice (Table S8). The control, UVB, and EU+UVB groups contained seven, one, and seven genera, respectively, with at least 18 nodes in the gut microbiota networks (Tables S9–S11). The co-occurrence network pattern changes indicated that chronic UVB exposure reduced the connectivity, robustness, and complexity of the gut microbial ecology in mice. Dietary EU enhanced the stability of the gut microbiota co-occurrence network, contributing to intestinal health in photoaged mice.

### 3.12. Analysis of the Correlation between the Skin Photoaging-Related Indexes, Hub Genes, and Relative Gut Microbial Abundance

**Figure 11:**
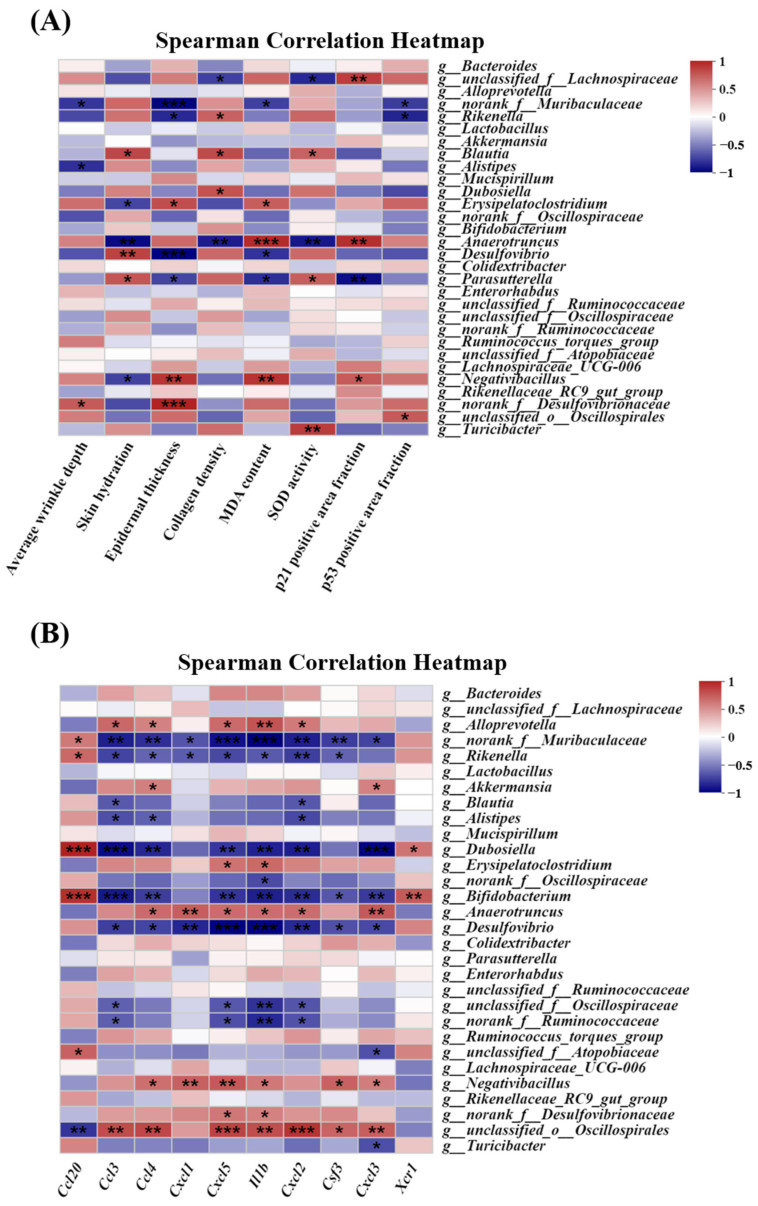
Correlation analysis among skin photoaging-related indexes, hub genes, and the relative abundance of gut microbiota at the genus level (top 30). (**A**) Correlation analysis between skin photoaging-related indexes and the relative abundance of gut microbiota. *n* = 3. (**B**) Correlation heatmap between hub gene expression determined by RNA-Seq and the relative abundance of gut microbiota. *n* = 5. Spearman correlation analysis was performed using the data from the control, UVB, and EU+UVB groups. The red represents a positive correlation, and the blue color indicates a negative correlation. Significant differences between groups are indicated as: * *p* < 0.05; ** *p* < 0.01; *** *p* < 0.001.

The correlation between the skin photoaging-related indexes, hub genes identified via RNA-seq analysis, and the relative gut microbial abundance at the genus level (top 30) was evaluated. Figure 11A shows the association between the skin photoaging-related indexes (average wrinkle depth, skin hydration, epidermal thickness, collagen density, MDA content, SOD activity, p21 positive area fraction, and p53 positive area fraction) and the relative abundance of gut microbiota at the genus level, as determined via Spearman correlation analysis. The results revealed a strong association between skin photoaging-related indexes and the relative abundance of gut microbiota. The change in the *Rikenella* relative abundance was positively associated with collagen density, and negatively with epidermal thickness and the p53 positive area fraction. The *Blautia* relative abundance variation was positively correlated with skin hydration, collagen density, and SOD activity, while the alteration in the *Dubosiella* relative abundance was positively associated with collagen density. The change in the *Erysipelatoclostridium* relative abundance was positively correlated with epidermal thickness and MDA content, and negatively with skin hydration. The *Anaerotruncus* relative abundance variation was positively related to the MDA content and p21 positive area fraction and negatively with skin hydration, collagen density, and SOD activity. The alteration in the *Desulfovibrio* relative abundance was positively associated with skin hydration, and negatively with epidermal thickness and MDA content. The *Parasutterella* relative abundance change was positively correlated with skin hydration and SOD activity and negatively with epidermal thickness, MDA content, and the p21 positive area fraction. The *Negativibacillus* relative abundance variation was positively related to epidermal thickness, MDA content, and the p21 positive area fraction and negatively with skin hydration. The alteration in the *Turicibacter* relative abundance was positively associated with SOD activity.

Figure 11B shows the correlation between the hub gene expression and the relative abundance of the gut microbiota, as determined via Spearman correlation analysis. The results revealed a significant correlation between the hub gene expression and the relative abundance of gut microbiota. The change in the *Alloprevotella* relative abundance was positively related to the *ccl3*, *ccl4*, *cxcl5*, *IL-1β*, and *cxcl2* levels. The *Rikenella* relative abundance variation was positively correlated with *ccl20* expression and negatively with the *ccl3*, *ccl4*, *cxcl1*, *cxcl5*, *IL-1β*, *cxcl2*, and *csf3* levels. The alteration in the *Akkermansia* relative abundance was positively related to *ccl4* and *cxcl3* expression, while the *Blautia* relative abundance change was negatively correlated with the *ccl3* and *cxcl2* levels. The *Alistipes* relative abundance variation was negatively associated with *ccl3*, *ccl4*, and *cxcl2* expression. The alteration in the *Dubosiella* relative abundance was positively related to the *ccl20* and *xcr1* levels and negatively with *ccl3*, *ccl4*, *cxcl5*, *IL-1β*, *cxcl2*, and *cxcl3* expression. The change in the *Erysipelatoclostridium* relative abundance was positively associated with the *cxcl5* and *IL-1β* levels. The *Bifidobacterium* relative abundance variation was positively related to *ccl20* and *xcr1* expression and negatively with the *ccl3*, *ccl4*, *cxcl5*, *IL-1β*, *cxcl2*, *csf3*, and *cxcl3* levels. The alteration in the *Anaerotruncus* relative abundance was positively associated with *ccl4*, *cxcl1*, *cxcl5*, *IL-1β*, *cxcl2*, and *cxcl3* expression, while the *Desulfovibrio* relative abundance change was negatively related to the *ccl3*, *ccl4*, *cxcl1*, *cxcl5*, *IL-1β*, *cxcl2*, *csf3*, and *cxcl3* levels. The *Negativibacillus* relative abundance variation was positively correlated with *ccl4*, *cxcl1*, *cxcl5*, *IL-1β*, *csf3*, and *cxcl3* expression, while The alteration in the *Turicibacter* relative abundance was negatively associated with the *cxcl3* level.

## 4. Discussion

Skin photoaging is considered an outside-inside mechanism due to the significant influence of UVB radiation. Damage affecting the epidermal layer not only causes epidermal aging but also extends downward, contributing to dermal aging [3,26]. After UVB exposure, the epidermal layer, which serves as the primary skin barrier, is the first to suffer damage. Adverse reactions include lower skin hydration levels and a dramatic decline in critical barrier protein expression, such as filaggrin, tight junction proteins, and keratins, disrupting the skin barrier [27]. Consequently, the equilibrium of the skin microenvironment is disrupted by a significant immune cell infiltration into the dermal layer and inflammatory factor release. The fibroblast cell populations, the major cells in the dermis, decrease to reduce ECM secretion, ultimately compromising structural skin integrity [28]. UVB exposure directly damages cellular DNA, leading to elevated expression of DNA damage marker p53 and the accumulation of DNA damage products in skin cells, exacerbating the dysregulation of the skin microenvironment [29]. Furthermore, UVB exposure causes substantial ROS accumulation in skin cells, triggering oxidative stress, disrupting normal cellular functions, and impairing skin regeneration capacity [30]. The present study demonstrated that EU alleviated skin photoaging by enhancing barrier repair (including improved skin hydration and upregulated barrier-protein-related gene expression), promoting tissue regeneration (increased ECM content and cell proliferation marker expression), and regulating the skin microenvironment (less oxidative damage, downregulated p21 and p53 expression, and inhibited SASP expression) (Figure 2, Figure 3, Figure 4 and Figure 5). This study proposes that EU may be a promising dietary supplement for preventing skin photoaging.

EU is Generally Recognized as Safe (GRAS) by the Flavor and Extract Manufacturers Association and has been approved as a flavoring agent by the U.S. Food and Drug Administration under 21 CFR 184.1257 [13]. The acute oral median lethal dose of EU has been determined as 3.0 g/kg in mice (strain and gender unspecified) [31]. The no observed adverse effect level of EU for mice has been established at 300 mg/kg body weight/day, as determined by a repeated-dose toxicity test [32]. Based on currently available data and usage levels, EU does not present concern regarding genetic toxicity [33]. The dosage administered to the mice in the present study was equivalent to a human dose of 0.24 g/day for an adult female (with an average weight of 59 kg) [34]. Our previous study showed that a dietary EU dosage of 200 mg/kg alleviated obesity in mice, with no observed deaths or adverse treatment-related clinical signs [15]. The EU dosage (50 mg/kg) administered to the mice in the present study was notably lower than the no-observed adverse effect level, as well as the dosages used in other studies. Furthermore, no adverse reactions were evident throughout the experimental process.

This study examined the EU distribution in the skin of mice to help clarify its potential benefits. The results showed EU content in skin at 0.06 μmol/kg (Table S3), suggesting that it reached the skin via the digestive and absorption processes, possibly exerting anti-photoaging effects. Research indicates that EU is rapidly absorbed and metabolized in the human body after oral administration, with nearly complete elimination through urine within 24 h [35]. Various EU metabolites were detected in the urine, including cis-isoEU, trans-isoEU, 3-(4-hydroxy-3-methoxyphenyl)-propylene-1,2-oxide, 3-(4-hydroxy-3-methoxyphenyl)-propane-1,2-diol, 3-(4-hydroxy-3-methoxyphenyl)-propionic acid, and 4-hydroxy-3-methoxyphenylpropane [35]. Only a small fraction, less than 0.1%, of EU is excreted unchanged in urine [35]. In rats, EU is also rapidly eliminated after oral administration, primarily through the urine [36]. Therefore, it is hypothesized that, besides EU itself, its metabolites potentially contribute to the beneficial effect on skin photoaging. Future pharmacokinetic EU studies on mice are necessary and crucial for a better understanding of its positive physiological effects.

Cell senescence is a key feature of aging [37]. Senescent cells develop SASPs, containing pro-inflammatory cytokines (IL-1, IL-6, IL-8, TNF-α, and IFN-γ), MMPs (MMP-1, MMP-3, MMP-9, and MMP-12), chemokines (CXCL1, CXCL2, CXCL5, CCCL5, and CCL7), growth factors (vascular endothelial growth factor and transforming growth factor), and nonprotein secretions such as ROS [38]. SASPs contribute significantly to skin photoaging [6]. One of the leading cytokines in the SASPs is proinflammatory IL-6, which is directly driven by UV-induced DNA damage in keratinocytes and fibroblasts [39]. Another notable interleukin upregulated by UV exposure is IL-1, with both IL-1α and IL-1β overexpressed by various photoaged skin cells [40]. The continued secretion of these inflammatory factors disrupts skin homeostasis, degrades the ECM, damages DNA, and exacerbates skin photoaging [6]. In addition to the secretion of some proinflammatory factors, UV-induced senescent cells also express enzymes for ECM remodeling, especially MMP1/3/9. These SASP-associated proteases greatly influence skin cell homeostasis by solubilizing membrane-associated proteins, breaking up and subsequently degenerating signaling molecules, processing, remodeling, or degrading the ECM [41]. Recent research has identified specific natural functional ingredients, such as rutin, that possess anti-aging properties, effectively delaying the aging process by selectively downregulating SASPs [42]. These substances are known as senomorphics and represent important tools in senotherapeutic strategies [43]. Consistent with previous research [44], the findings of this study demonstrated that chronic UVB exposure increased SASP expression in mouse skin (Figure 7C–P). Dietary EU exhibits consistency in mitigating skin photoaging and inhibiting chronic UV-induced elevation of SASPs (Figure 7C–P), indicating that EU shows promise for the development of senomorphic agents. However, additional investigations are required to validate the senomorphic effect of EU.

Chronic UVB exposure reportedly induces common gene expression signatures in skin tissues or cells, which can be used to predict the anti-photoaging effect of new candidate compounds. For example, Yan et al. performed a KEGG enrichment analysis, identifying ECM–receptor interaction as an upregulated functional module in the sun-exposed pre-auricular skin of Chinese women. They hypothesized that ECM–receptor interaction acted as the TGF-β family signaling pathway for cell cycle and apoptosis regulation in the skin [45]. During skin photoaging, upregulated and activated TGF-β produced excessive MMPs and pro-inflammatory cytokines, prolonging neutrophil infiltration and the progressive degradation of collagen, HA, and elastic fibers, which contributed to ECM destruction [46]. Furthermore, KEGG enrichment analysis by Alafiatayo et al. revealed significant upregulation in the cytokine–cytokine receptor interaction in UV-exposed fibroblasts [47]. The present study further characterized the biological processes and signaling pathways enriched in the samples using gene enrichment analysis. Both KEGG analysis and GSEA revealed that dietary EU supplementation effectively reversed the upregulation of the cytokine–cytokine receptor interaction and ECM–receptor interaction in the skin of photoaged mice (Figure 6H,M,O), indicating that EU regulated the common signaling pathways during the skin photoaging processes.

Studies have demonstrated that the composition, structure, and functionality of gut microbiota are closely associated with skin aging [7,48]. With age progression, a discernible decline is evident in the beneficial anti-inflammatory intestinal bacterial species in mice, such as *Faecalibacterium* and *Bifidobacteria* [49]. Furthermore, research involving humans reveals a transition from the Firmicutes phyla to an elevation in the Bacteroidetes phyla from adulthood to old age [50]. Dietary probiotics intervention reportedly ameliorates various manifestations of skin aging, such as impaired skin barrier function, photodamage, oxidative stress, and acidic skin pH [51]. Ghaly et al. showed that ultraviolet (UV) skin irradiation altered the fecal microbiome in female C57BL/6 mice [52]. Our previous study demonstrated that chronic skin exposure to UVB triggered gut microbiota disruption and skin photoaging in male C57BL/6 mice [53]. The present study indicated that chronically exposing skin to UVB caused skin photoaging and gut microbiota disorders in female SKH-1 hairless mice. Furthermore, dietary EU ameliorated both skin photodamage and intestinal bacterial dysbiosis. The current study indicated that the ameliorative effect of EU on skin photoaging may be linked to its mitigation of intestinal microbial disturbances.

The characteristic gut bacteria, *Rikenellaceae_RC9_gut_group* and *Mucispirillum*, identified via LEFSe and ANCOM analyses (Figure 9A,B and Table S6), reportedly exhibit significantly higher abundance in the gut microbiota of mice afflicted with various dermatological conditions. For instance, Ghaly et al. noted a substantial rise in the *Mucispirillum* relative abundance in the gut microbiota of C57BL/6 mice after chronic UVB skin exposure [52], showing notable upregulation in mice with atopic dermatitis [54,55]. Furthermore, the gut microbiota of skin-aged rats induced by D-galactose showed elevated *Mucispirillum* relative abundance, which was effectively reduced by *Sacha inchi* albumin, ameliorating skin aging [56]. The relative abundance of *Rikenellaceae_RC9_gut_group* was notably upregulated in oleic acid-induced cutaneous acne mice, exhibiting a positive correlation with the inflammatory factor levels [57]. Consistent with the reports mentioned above, the findings of the present study confirmed the increased abundance of *Rikenellaceae_RC9_gut_group* and *Mucispirillum* in the feces of photoaged mice, which was significantly reduced by dietary EU supplementation (Figure 9C and Table S7), underscoring their potential importance in the efficacy of EU to mitigate gut microbiota dysbiosis. However, investigations into the regulatory roles of *Rikenellaceae_RC9_gut_group* and *Mucispirillum* during the skin photoaging process remain limited.

The present study employed female SKH-1 hairless mice to explore the preventative effect of EU on skin photoaging and its underlying mechanisms. Research has shown that gender plays a crucial role in the onset of various diseases, with variations in how females and males respond to identical stimuli (e.g., UV exposure) [58]. Reeve et al. found that male SKH-1 hairless mice exhibited a relatively delayed response to the photoimmune effect of single UV exposure (including UVA and UVB). In equivalent UV exposure conditions, male mice exhibited significantly lower inflammatory factor expression levels in their skin compared to female mice [59]. However, Zhong et al. reported that male Kunming mice exhibited a higher sensitivity to chronic UV radiation (UVA:UVB = 93:7) than their female counterparts, displaying more severe inflammatory damage and oxidative stress in their skin [60]. The differential responses of mice to skin photodamage and dietary interventions caused by gender differences are also of interest to our future research. Moreover, distinct structural differences are evident between the skin of humans and mice. For instance, the stratum corneum of mice tends to be more susceptible to damage and allows for more significant permeability compared to humans [61]. In mice, melanocytes are predominantly located in the dermis and hair follicles, while they are primarily situated in the basal layer of the epidermis in humans [62]. Therefore, further research is necessary to investigate the potential protective effect of dietary EU supplementation on skin photoaging in humans.

## 5. Conclusions

In summary, EU effectively mitigates the effect of UVB-induced skin photoaging in vitro and in vivo. EU alleviates UVB exposure-induced photodamage in HaCaT epidermal keratinocytes and Hs68 dermal fibroblasts by mitigating oxidative stress and decreasing the ECM. Dietary EU supplementation restores the skin barrier, facilitates tissue regeneration, and modulates the skin microenvironment in photoaged mice. Transcriptomic sequencing analysis of the skin underscores the profound impact of dietary EU on a multitude of signaling pathways and physiological processes pertinent to skin health and related conditions. The identification of cytokine–cytokine receptor interaction and ECM–receptor interaction as pivotal pathways reversed by EU intervention highlights their essential roles in ameliorating the detrimental consequences of prolonged UVB exposure on skin aging. Furthermore, 16S rRNA sequencing analysis reveals that dietary EU effectively mitigates intestinal dysbiosis caused by chronic UVB exposure. Further correlation analysis indicates a close association between gut microbiota and skin photoaging-related parameters, as well as the hub genes in the key signaling pathways. The results emphasize the potential of EU as a viable approach for preventing UVB-induced skin photoaging, providing alternative strategies for reverting extrinsic skin aging.

## Data Availability

The data that support the findings of this study are available from the corresponding author upon reasonable request.

## Supplementary Materials

The following supporting information can be downloaded at: https://www.mdpi.com/article/10.3390/antiox13020168/s1, Figure S1: EU treatment appears to have no significant effect on normal HaCaT and Hs68 cells; Figure S2: The effects of dietary supplementation with eugenol on body weight and food intake in chronic UVB-exposed mice; Table S1: Composition of the experimental diets (g/kg) fed to mice; Table S2: Primer sequences used for RT-qPCR; Table S3: Eugenol accumulation in the skin of eugenol group mice; Table S4: TOP50 GSEA enrichment results based on all genes in the UVB group compared to the control group (*p*-value < 0.05); Table S5: TOP50 GSEA enrichment results based on all genes in the eugenol group compared to the UVB group (*p*-value < 0.05); Table S6: The ANCOM analysis of gut microbiota at genus level; Table S7: Abundance of gut microbial strains identified by LEFSe and ANCOM analysis at the genus level; Table S8: Global network parameters; Table S9: Description of taxa information for each node in the control group network; Table S10: Description of taxa information for each node in the UVB group network; Table S11: Description of taxa information for each node in the eugenol group network.

## References

[1] Geng R., Wang Y., Fang J., Zhao Y., Li M., Kang S.G., Huang K., Tong T. (2022). Ectopic odorant receptors responding to flavor compounds in skin health and disease: Current insights and future perspectives. Crit. Rev. Food Sci..

[2] Franco A.C., Aveleira C., Cavadas C. (2022). Skin senescence: Mechanisms and impact on whole-body aging. Trends Mol. Med..

[3] Krutmann J., Schikowski T., Morita A., Berneburg M. (2021). Environmentally-induced (extrinsic) skin aging: Exposomal factors and underlying mechanisms. J. Investig. Dermatol..

[4] Debacq-Chainiaux F., Leduc C., Verbeke A., Toussaint O. (2012). UV, stress and aging. Derm.-Endocrinol..

[5] Huang A.H., Chien A.L. (2020). Photoaging: A review of current literature. Curr. Dermatol. Rep..

[6] Bulbiankova D., Diaz-Puertas R., Alvarez-Martinez F.J., Herranz-Lopez M., Barrajon-Catalan E., Micol V. (2023). Hallmarks and biomarkers of skin senescence: An updated review of skin senotherapeutics. Antioxidants.

[7] Geng R., Kang S., Huang K., Tong T. (2021). Boosting the photoaged skin: The potential role of dietary components. Nutrients.

[8] Tanveer M.A., Rashid H., Tasduq S.A. (2023). Molecular basis of skin photoaging and therapeutic interventions by plant-derived natural product ingredients: A comprehensive review. Heliyon.

[9] Faria-Silva C., Ascenso A., Costa A.M., Marto J., Carvalheiro M., Ribeiro H.M., Simões S. (2020). Feeding the skin: A new trend in food and cosmetics convergence. Trends Food Sci. Technol..

[10] Kumar P.V., Shams R., Singh R., Dar A.H., Pandiselvam R., Rusu A.V., Trif M. (2022). A comprehensive review on clove (*Caryophyllus aromaticus* L.) essential oil and its significance in the formulation of edible coatings for potential food applications. Front. Nutr..

[11] Du P., Yuan H., Chen Y., Zhou H., Zhang Y., Huang M., Jiangfang Y., Su R., Chen Q., Lai J. (2023). Identification of key aromatic compounds in basil (*Ocimum* L.) using sensory evaluation, metabolomics and volatilomics analysis. Metabolites.

[12] Yadav M.K., Chae S.W., Im G.J., Chung J.W., Song J.J. (2015). Eugenol: A phyto-compound effective against methicillin-resistant and methicillin-sensitive staphylococcus aureus clinical strain biofilms. PLoS ONE.

[13] Nisar M.F., Khadim M., Rafiq M., Chen J., Yang Y., Wan C.C. (2021). Pharmacological properties and health benefits of eugenol: A comprehensive review. Oxidative Med. Cell. Longev..

[14] Pramod K., Ansari S.H., Ali J. (2010). Eugenol: A natural compound with versatile pharmacological actions. Nat. Prod. Commun..

[15] Li M., Zhao Y., Wang Y., Geng R., Fang J., Kang S.G., Huang K., Tong T. (2022). Eugenol, a major component of clove oil, attenuates adiposity, and modulates gut microbiota in high-fat diet-fed mice. Mol. Nutr. Food Res..

[16] Ashjazadeh M.A., Jahandideh A., Abedi G., Akbarzadeh A., Hesaraki S. (2019). Histopathology and histomorphological study of wound healing using clove extract nanofibers (eugenol) compared to zinc oxide nanofibers on the skin of rats. Arch. Razi Inst..

[17] De Araujo L.A., Da F.F., Rocha T.M., de Freitas L.B., Araujo E., Wong D., Lima J.R., Leal L. (2018). Eugenol as a promising molecule for the treatment of dermatitis: Antioxidant and anti-inflammatory activities and its nanoformulation. Oxidative Med. Cell. Longev..

[18] Pal D., Banerjee S., Mukherjee S., Roy A., Panda C.K., Das S. (2010). Eugenol restricts DMBA croton oil induced skin carcinogenesis in mice: Downregulation of c-Myc and H-ras, and activation of p53 dependent apoptotic pathway. J. Dermatol. Sci..

[19] Kamatou G.P., Vermaak I., Viljoen A.M. (2012). Eugenol—From the remote Maluku Islands to the international market place: A review of a remarkable and versatile molecule. Molecules.

[20] Hwang E., Lin P., Ngo H., Yi T.H. (2018). Clove attenuates UVB-induced photodamage and repairs skin barrier function in hairless mice. Food Funct..

[21] Yarosh D.B., Yee V. (1990). SKH-1 hairless mice repair UV-induced pyrimidine dimers in epidermal DNA. J. Photochem. Photobiol. B Biol..

[22] Kang W., Choi D., Park T. (2019). Dietary suberic acid protects against UVB-induced skin photoaging in hairless mice. Nutrients.

[23] Geng R., Kang S., Huang K., Tong T. (2022). A-ionone protects against UVB-induced photoaging in epidermal keratinocytes. Chin. Herb. Med..

[24] Subramanian A., Tamayo P., Mootha V.K., Mukherjee S., Ebert B.L., Gillette M.A., Paulovich A., Pomeroy S.L., Golub T.R., Lander E.S. (2005). Gene set enrichment analysis: A knowledge-based approach for interpreting genome-wide expression profiles. Proc. Natl. Acad. Sci. USA.

[25] Nearing J.T., Douglas G.M., Hayes M.G., MacDonald J., Desai D.K., Allward N., Jones C., Wright R.J., Dhanani A.S., Comeau A.M. (2022). Microbiome differential abundance methods produce different results across 38 datasets. Nat. Commun..

[26] Fisher G.J., Wang Z.Q., Datta S.C., Varani J., Kang S., Voorhees J.J. (1997). Pathophysiology of premature skin aging induced by ultraviolet light. N. Engl. J. Med..

[27] Park N.J., Bong S.K., Lee S., Jung Y., Jegal H., Kim J., Kim S.K., Kim Y.K., Kim S.N. (2020). Compound K improves skin barrier function by increasing SPINK5 expression. J. Ginseng Res..

[28] Hernandez-Barrera R., Torres-Alvarez B., Castanedo-Cazares J.P., Oros-Ovalle C., Moncada B. (2008). Solar elastosis and presence of mast cells as key features in the pathogenesis of melasma. Clin. Exp. Dermatol..

[29] Carpenter E.L., Le M.N., Miranda C.L., Reed R.L., Stevens J.F., Indra A.K., Ganguli-Indra G. (2018). Photoprotective properties of isothiocyanate and nitrile glucosinolate derivatives from meadowfoam (*Limnanthes alba*) against UVB irradiation in human skin equivalent. Front. Pharmacol..

[30] Yang Z., Dong L., Jin S., Han X., Li F. (2023). Comparison of microfat, nanofat, and extracellular matrix/stromal vascular fraction gel for skin rejuvenation: Basic animal research. Aesthet. Surg. J..

[31] National-Toxicology-Program (1983). Carcinogenesis studies of eugenol (CAS No. 97-53-0) in F344/N rats and B6C3F1 mice (feed studies). Natl. Toxicol. Program Tech. Rep. Ser..

[32] Api A.M., Belsito D., Bhatia S., Bruze M., Calow P., Dagli M.L., Dekant W., Fryer A.D., Kromidas L., La Cava S. (2016). RIFM fragrance ingredient safety assessment, eugenol, CAS registry number 97-53-0. Food Chem. Toxicol..

[33] Api A.M., Belsito D., Botelho D., Bruze M., Burton G.J., Buschmann J., Cancellieri M.A., Dagli M.L., Date M., Dekant W. (2022). Update to RIFM fragrance ingredient safety assessment, eugenol, CAS registry number 97-53-0. Food Chem. Toxicol..

[34] Reagan-Shaw S., Nihal M., Ahmad N. (2008). Dose translation from animal to human studies revisited. FASEB J. Off. Publ. Fed. Am. Soc. Exp. Biol..

[35] Fischer I.U., von Unruh G.E., Dengler H.J. (1990). The metabolism of eugenol in man. Xenobiotica.

[36] Sutton J.D., Sangster S.A., Caldwell J. (1985). Dose-dependent variation in the disposition of eugenol in the rat. Biochem. Pharmacol..

[37] LópezOtín C., Blasco M.A., Partridge L., Serrano M., Kroemer G. (2022). Hallmarks of aging: An expanding universe. Cell.

[38] Singh P., Gollapalli K., Mangiola S., Schranner D., Yusuf M.A., Chamoli M., Shi S.L., Lopes B.B., Nair T., Riermeier A. (2023). Taurine deficiency as a driver of aging. Science.

[39] Lu S.Y., Chang K.W., Liu C.J., Tseng Y.H., Lu H.H., Lee S.Y., Lin S.C. (2006). Ripe areca nut extract induces G1 phase arrests and senescence-associated phenotypes in normal human oral keratinocyte. Carcinogenesis.

[40] Ozcan S., Alessio N., Acar M.B., Mert E., Omerli F., Peluso G., Galderisi U. (2016). Unbiased analysis of senescence associated secretory phenotype (SASP) to identify common components following different genotoxic stresses. Aging.

[41] Freitas-Rodriguez S., Folgueras A.R., Lopez-Otin C. (2017). The role of matrix metalloproteinases in aging: Tissue remodeling and beyond. Biochim. Biophys. Acta Mol. Cell Res..

[42] Liu H., Xu Q., Wufuer H., Li Z., Sun R., Jiang Z., Dou X., Fu Q., Campisi J., Sun Y. (2023). Rutin is a potent senomorphic agent to target senescent cells and can improve chemotherapeutic efficacy. Aging Cell.

[43] Di Micco R., Krizhanovsky V., Baker D., D’Adda D.F.F. (2021). Cellular senescence in ageing: From mechanisms to therapeutic opportunities. Nat. Rev. Mol. Cell Biol..

[44] Kim H., Jang J., Song M.J., Park C.H., Lee D.H., Lee S.H., Chung J.H. (2022). Inhibition of matrix metalloproteinase expression by selective clearing of senescent dermal fibroblasts attenuates ultraviolet-induced photoaging. Biomed. Pharmacother..

[45] Yan W., Zhang L.L., Yan L., Zhang F., Yin N.B., Lin H.B., Huang C.Y., Wang L., Yu J., Wang D.M. (2013). Transcriptome analysis of skin photoaging in chinese females reveals the involvement of skin homeostasis and metabolic changes. PLoS ONE.

[46] Ke Y., Wang X.J. (2021). TGFbeta signaling in photoaging and UV-induced skin cancer. J. Investig. Dermatol..

[47] Alafiatayo A.A., Lai K.S., Ahmad S., Mahmood M., Shaharuddin N.A. (2020). RNA-Seq analysis revealed genes associated with UV-induced cell necrosis through MAPK/TNF-alpha pathways in human dermal fibroblast cells as an inducer of premature photoaging. Genomics.

[48] Boyajian J.L., Ghebretatios M., Schaly S., Islam P., Prakash S. (2021). Microbiome and human aging: Probiotic and prebiotic potentials in longevity, skin health and cellular senescence. Nutrients.

[49] Thevaranjan N., Puchta A., Schulz C., Naidoo A., Szamosi J.C., Verschoor C.P., Loukov D., Schenck L.P., Jury J., Foley K.P. (2018). Age-associated microbial dysbiosis promotes intestinal permeability, systemic inflammation, and macrophage dysfunction. Cell Host Microbe.

[50] Vaiserman A., Romanenko M., Piven L., Moseiko V., Lushchak O., Kryzhanovska N., Guryanov V., Koliada A. (2020). Differences in the gut Firmicutes to Bacteroidetes ratio across age groups in healthy Ukrainian population. BMC Microbiol..

[51] Ratanapokasatit Y., Laisuan W., Rattananukrom T., Petchlorlian A., Thaipisuttikul I., Sompornrattanaphan M. (2022). How microbiomes affect skin aging: The updated evidence and current perspectives. Life.

[52] Ghaly S., Kaakoush N.O., Lloyd F., Gordon L., Forest C., Lawrance I.C., Hart P.H. (2018). Ultraviolet irradiation of skin alters the faecal microbiome independently of vitamin d in mice. Nutrients.

[53] Geng R., Fang J., Kang S.G., Huang K., Tong T. (2023). Chronic exposure to UVB induces nephritis and gut microbiota dysbiosis in mice based on the integration of renal transcriptome profiles and 16S rRNA sequencing data. Environ. Pollut..

[54] Jiang X., Liu Z., Ma Y., Miao L., Zhao K., Wang D., Wang M., Ruan H., Xu F., Zhou Q. (2023). Fecal microbiota transplantation affects the recovery of AD-skin lesions and enhances gut microbiota homeostasis. Int. Immunopharmacol..

[55] Kim W.K., Jang Y.J., Han D.H., Seo B., Park S., Lee C.H., Ko G. (2019). Administration of lactobacillus fermentum KBL375 causes taxonomic and functional changes in gut microbiota leading to improvement of atopic dermatitis. Front. Mol. Biosci..

[56] Zeng J., An M., Tian W., Wang K., Du B., Li P. (2023). Sacha inchi albumin delays skin-aging by alleviating inflammation, oxidative stress and regulating gut microbiota in d-galactose induced-aging mice. J. Sci. Food Agric..

[57] Ai J., Ma W., Pan Z., Mao B., Tang X., Zhang Q., Zhao J., Chen W., Cui S. (2023). Ameliorative effect of *Lactobacillus plantarum* CCFM8661 on oleic acid-induced acne: Integrated gut microbiota link to acne pathogenesis. J. Sci. Food Agric..

[58] Skopelja-Gardner S., Tai J., Sun X., Tanaka L., Kuchenbecker J.A., Snyder J.M., Kubes P., Mustelin T., Elkon K.B. (2021). Acute skin exposure to ultraviolet light triggers neutrophil-mediated kidney inflammation. Proc. Natl. Acad. Sci. USA.

[59] Reeve V.E., Allanson M., Domanski D., Painter N. (2012). Gender differences in UV-induced inflammation and immunosuppression in mice reveal male unresponsiveness to UVA radiation. Photoch. Photobio. Sci..

[60] Zhong Q.Y., Lin B., Chen Y.T., Huang Y.P., Feng W.P., Wu Y., Long G.H., Zou Y.N., Liu Y., Lin B.Q. (2021). Gender differences in UV-induced skin inflammation, skin carcinogenesis and systemic damage. Environ. Toxicol. Pharmacol..

[61] Simon G., Dana B., Jacqueline C., Michael W.C., Anderson P.J., Stephanie T., Allan G.K., Rachel E.N., Prue H.H. (2018). Vitamin D C3-epimer levels are proportionally higher with oral vitamin D supplementation compared to ultraviolet irradiation of skin in mice but not humans. J. Steroid Biochem..

[62] Slominski A.T., Zmijewski M.A., Plonka P.M., Szaflarski J.P., Paus R. (2018). How UV light touches the brain and endocrine system through skin, and why. Endocrinology.

