# Supplementary material for: Revitalizing Photoaging Skin through Eugenol in UVB-Exposed Hairless Mice: Mechanistic Insights from Integrated Multi-Omics
Source: Antioxidants (Basel). 2024 Jan 29;13(2):168. doi: 10.3390/antiox13020168 (PMC10886361; doi:10.3390/antiox13020168)
Supplement: Supplementary file 1 [file antioxidants-13-00168-s001.zip › antioxidants-2803035-supplementary.pdf]

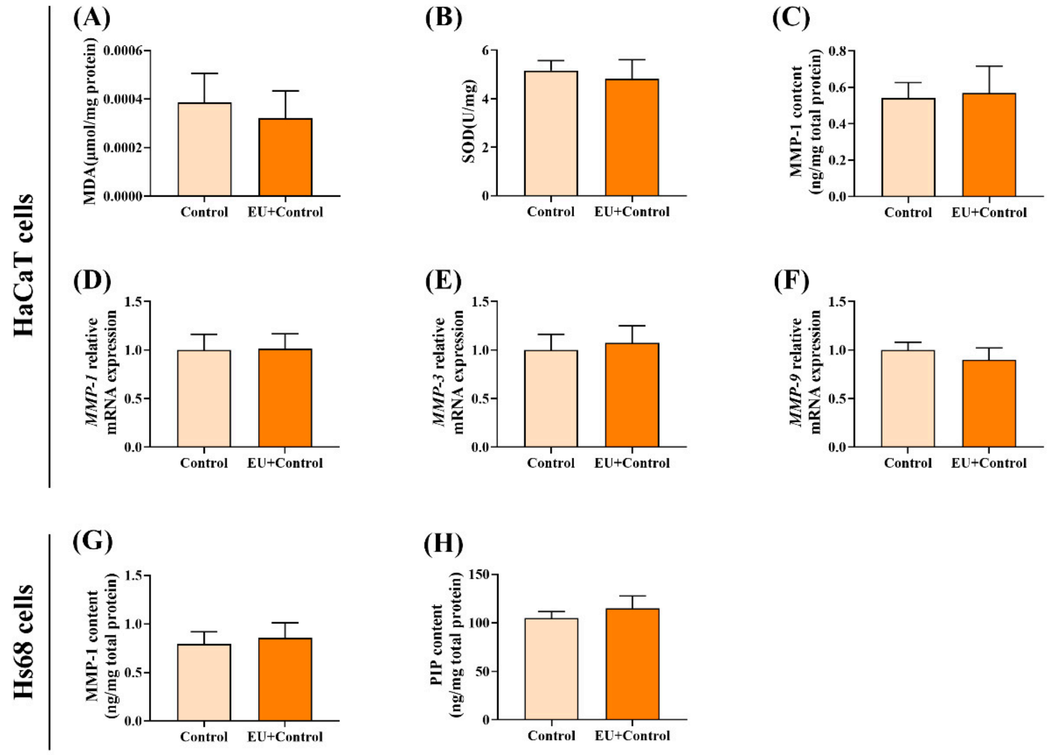

Figure S1. EU treatment appears to have no significant effect on normal HaCaT and Hs68 cells. (A) MDA content in HaCaT cells. (B) SOD level in HaCaT cells. (C) MMP-1 content in HaCaT cells. (D) Relative mRNA expression of *MMP-1* in HaCaT cells. (E) Relative mRNA expression of *MMP-3* in HaCaT cells. (F) Relative mRNA expression of *MMP-9* in HaCaT cells. (G) MMP-1 content in Hs68 cells. (H) PIP content in Hs68 cells. Data were presented as mean ± SEM (n = 3).

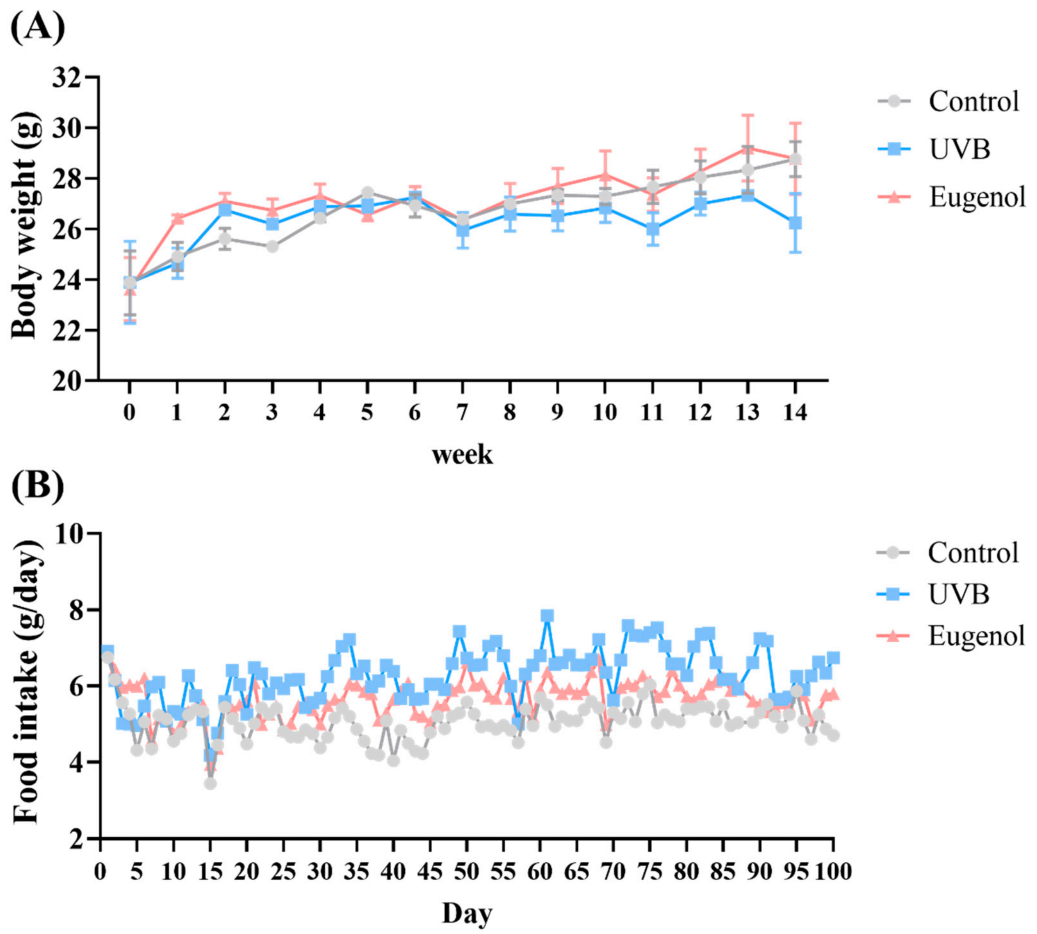

Figure S2. The effects of dietary supplementation with EU on body weight and food intake in chronic UVB-exposed mice. (A) Body weight. (B) Food intake. Data were presented as mean ± SEM (n = 7).

Table S1. Composition of the experimental diets (g/kg) fed to mice.

Ingredients	Control and UVB groups (AIN93G diet)	EU+UVB group (AIN93G diet supplemented with 0.05% EU)
Corn starch	397.486	396.986
Casein	200	200
Maltodextrin	132	132
Sucrose	100	100
Soybean oil	70	70
Cellulose	50	50
Mineral mix[1]	35	35
Vitamin mix[2]	10	10
L-Cystine	3	3
Choline bitartrate	2.5	2.5
tert-Butylhydroquinone	0.014	0.014
EU	-	0.5

[1] Mineral mix: AIN93G–Mineral mix.

[2] Vitamin mix: AIN93G–Vitamin mix.

Table S2. Primer sequences used for RT-qPCR.

Type	Gene Description	Sequences (5'→3')
Human	*β-Actin*	F: AGCGAGCATCCCCCAAAGTT<br>R: GGGCACGAAGGCTCATCATT
	Matrix metalloproteinase-1 (*MMP-1*)	F: ATTCTACTGATATCGGGGCTTTGA<br>R: ATGTCCTTGGGGTATCCGTGTAG
	Matrix metalloproteinase-3 (*MMP-3*)	F: TGAGGACACCAGCATGAACC<br>R: ACTTCGGATGCCCAGGAAAG
	Matrix metalloproteinase-9 (*MMP-9*)	F: TTGACAGCGACAAGAAGTGG<br>R: GCCATTCACGTCGTCCTTAT
Mouse	*β-Actin*	F: GTCCCTCACCCTCCCAAAAG<br>R: GCTGCCTCAACACCTCAACCC
	β-Catenin	F: GGCGGCCGCGAGGTA<br>R: TTAGTGGGATGAGCAGCGTC
	Zonula occludens-1 (*ZO-1*)	F: AGTTCTGCCCTCAGCTACCA<br>R: GCTTAAAGCTGGCAGTGTC
	Keratin 1	F: GGAGGCTTCTGTGGGAGTTC<br>R: AAGTTTTGGGTCCGGGTTGT
	Keratin 10	F: GCCTACCTAAAGAAGAACCACGAA<br>R: GCAGCGTTCATTTCCACATTC
	Filaggrin	F: TGCATCTCCAGGTATATCGTTTCTT<br>R: GAACTATCACCCAAATGGAAAAACC
	Collagen type I alpha 1 chain (*COL1A1*)	F: GGCAACAGTCGCTTCACCTA<br>R: AGTCCGAATTCCTGGTCTGG
	Collagen type I alpha 2 chain (*COL1A2*)	F: CCCAGAGTGGAACAGCGATT<br>R: ATGAGTTCTTCGCTGGGGTG
	Collagen type III alpha 1 chain (*COL3A1*)	F: TAACCAAGGCTGCAAGATGG<br>R: ACCAGTGCTTACGTGGGACA
	Hyaluronic acid synthases 1 (*HAS1*)	F: CTATGCTACCAAGTATACCTCG<br>R: TCTCGGAAGTAAGATTTGGAC
	Hyaluronic acid synthases 2 (*HAS2*)	F: CGGTCGTCTCAAATTCATCTG<br>R: ACAATGCATCTTGTTCAGCTC
	Hyaluronic acid synthases 3 (*HAS3*)	F: GATGTCCAAATCCTCAACAAG<br>R: CCCACTAATACATTGCACAC
	Ki67	F: GCCTCCTAATACACCACTGAA<br>R: GCCGTTCCTTGATGATTGTC
	Proliferating cell nuclear antigen (*PCNA*)	F: GAGCAACTTGGAATCCCAGAACAG<br>R: CCAAGCTCCCCACTCGCAGAAAACT

MMP-1	F: AAGGTTAGCTTACTGTCACACGCTT<br>R: CGACTCTAGAAACACAAGAGCAAGA
MMP-3	F: ACTCCCTGGGACTCTACCAC<br>R: GGTACCACGAGGACATCAGG
MMP-9	F: GTGGACCATGAGGTGAACCA<br>R: ACTGCACGGTTGAAGCAAAG
Matrix metalloproteinase-13 (*MMP-13*)	F: CATCCATCCCGTGACCTTAT<br>R: GCATGACTCTCACAATGCGA
Interleukin-1β (*IL-1β*)	F: GCAACTGTTCCTGAACTCAACT<br>R: ATCTTTTGGGGTCCGTCAACT
Interleukin-6 (*IL-6*)	F: CTGCAAGAGACTTCCATCCAGTT<br>R: GAAGTAGGGAAGGCCGTGG
Tumor necrosis factor-α (*TNF-α*)	F: CAGGCGGTGCCTATGTCTC<br>R: CGATCACCCCGAAGTTCAGTAG
Interferon-γ (*IFN-γ*)	F: GCTCTGAGACAATGAACGCTACA<br>R: TTTCTTCCACATCTATGCCACTT
Monocyte chemoattractant protein-1 (*MCP-1*)	F: GGCTGTAGCCAAACTAGGCA<br>R: GCCATGGGTTAGGCAGAGTT
C-X-C motif chemokine 2 (*CXCL2*)	F: AGTGAACTGCGCTGTCAATG<br>R: TCCAGGTCAGTTAGCCTTGC
C-X-C motif chemokine 5 (*CXCL5*)	F: GCCCTACGGTGGAAGTCATA<br>R: GTGCATTCGCTTAGCTTTC
C-C motif ligand 5 (*CCL5*)	F: CCTGCTGCTTTGCCTACCTCTC<br>R: ACACACTTGGCGGTTCCTTCGA
C-C motif ligand 7 (*CCL7*)	F: CAGAAGGATCACCAGTAGTCGG<br>R: ATAGCCTCCTCGACCCACTTCT
Brain derived neurotrophic factor (*BDNF*)	F: TTGTTTTGTGCCGTTTACCA<br>R: GGTAAGAGAGCCAGCCACTG

Table S3. EU accumulation in the skin of EU+UVB group mice.

Sample	Concentration (μmol/kg)
Skin	0.06 ± 0.02

Data was presented as mean ± SEM, *n* = 3.

Table S4. TOP50 GSEA enrichment results based on all genes in the UVB group compared to the control group (*p*-value < 0.05).

Gene set name	Description	NES	*p*
KEGG_RIBOSOME	Ribosome	1.93	0.0000
KEGG_DNA_REPLICATION	DNA replication	1.78	0.0000
KEGG_ECM_RECEPTOR_INTERACTION	ECM-receptor interaction	1.72	0.0000
KEGG_CYTOSOLIC_DNA_SENSING_PATHWAY	Cytosolic DNA-sensing pathway	1.70	0.0000
KEGG_PROTEASOME	Proteasome	1.62	0.0000
KEGG_LEISHMANIA_INFECTION	Leishmania infection	1.55	0.0056
KEGG_P53_SIGNALING_PATHWAY	p53 signaling pathway	1.53	0.0000
KEGG_TOLL_LIKE_RECEPTOR_SIGNALING_PATHWAY	Toll-like receptor signaling pathway	1.51	0.0000
KEGG_CHEMOKINE_SIGNALING_PATHWAY	Chemokine signaling pathway	1.51	0.0000
KEGG_PEROXISOME	Peroxisome	-1.49	0.0035
KEGG_LYSOSOME	Lysosome	1.49	0.0000
KEGG_PYRIMIDINE_METABOLISM	Pyrimidine metabolism	1.48	0.0101
KEGG_PPAR_SIGNALING_PATHWAY	PPAR signaling pathway	-1.47	0.0047
KEGG_VALINE_LEUCINE_AND_ISOLEUCINE_DEGRADATION	Valine, leucine and isoleucine degradation	-1.43	0.0138
KEGG_RNA_POLYMERASE	RNA polymerase	1.43	0.0381
KEGG_FOCAL_ADHESION	Focal adhesion	1.42	0.0000
KEGG_FC_GAMMA_R_MEDIATED_PHAGOCYTOSIS	Fc gamma R-mediated phagocytosis	1.42	0.0000
KEGG_BIOSYNTHESIS_OF_UNSATURATED_FATTY_ACIDS	Biosynthesis of unsaturated fatty acids	-1.39	0.0331
KEGG_CYTOKINE_CYTOKINE_RECEPTOR_INTERACTION	Cytokine-cytokine receptor interaction	1.35	0.0000
KEGG_CELL_CYCLE	Cell cycle	1.35	0.0000
KEGG_B_CELL_RECEPTOR_SIGNALING_PATHWAY	B cell receptor signaling pathway	1.33	0.0240
KEGG_REGULATION_OF_ACTIN_CYTOSKELETON	Regulation of actin cytoskeleton	1.27	0.0000
KEGG_PURINE_METABOLISM	Purine metabolism	1.23	0.0408
KEGG_CELL_ADHESION_MOLECULES_CAMS	Cell adhesion molecules (CAMs)	1.17	0.0472

Abbreviations: GSEA, gene set enrichment analysis; NES, normalized enrichment score.

Table S5. TOP50 GSEA enrichment results based on all genes in the EU+UVB group compared to the UVB group (*p*-value < 0.05).

Gene set name	Description	NES	*p*
KEGG_ECM_RECEPTOR_INTERACTION	ECM-receptor interaction	-1.76	0.0000
KEGG_PEROXISOME	Peroxisome	1.68	0.0000
KEGG_VALINE_LEUCINE_AND_ISOLEUCINE_DEGRADATION	Valine, leucine and isoleucine degradation	1.64	0.0000
KEGG_FOCAL_ADHESION	Focal adhesion	-1.59	0.0000
KEGG_RETINOL_METABOLISM	Retinol metabolism	1.51	0.0054
KEGG_BIOSYNTHESIS_OF_UNSATURATED_FATTY_ACIDS	Biosynthesis of unsaturated fatty acids	1.50	0.0150
KEGG_TERPENOID_BACKBONE_BIOSYNTHESIS	Terpenoid backbone biosynthesis	1.50	0.0177
KEGG_FATTY_ACID_METABOLISM	Fatty acid metabolism	1.50	0.0097
KEGG_GAP_JUNCTION	Gap junction	-1.48	0.0000
KEGG_LEISHMANIA_INFECTION	Leishmania infection	-1.45	0.0149
KEGG_TRYPTOPHAN_METABOLISM	Tryptophan metabolism	1.45	0.0162
KEGG_BUTANOATE_METABOLISM	Butanoate metabolism	1.45	0.0247
KEGG_REGULATION_OF_ACTIN_CYTOSKELETON	Regulation of actin cytoskeleton	-1.42	0.0000
KEGG_DILATED_CARDIOMYOPATHY	Dilated cardiomyopathy	-1.41	0.0221
KEGG_STEROID_HORMONE_BIOSYNTHESIS	Steroid hormone biosynthesis	1.41	0.0316
KEGG_PROPANOATE_METABOLISM	Propanoate metabolism	1.41	0.0274
KEGG_COMPLEMENT_AND_COAGULATION_CASCADES	Complement and coagulation cascades	-1.40	0.0200
KEGG_CYTOSOLIC_DNA_SENSING_PATHWAY	Cytosolic DNA-sensing pathway	-1.39	0.0283
KEGG_ANTIGEN_PROCESSING_AND_PRESENTATION	Antigen processing and presentation	1.39	0.0319
KEGG_PPAR_SIGNALING_PATHWAY	PPAR signaling pathway	1.35	0.0406
KEGG_HYPERTROPHIC_CARDIOMYOPATHY_HCM	Hypertrophic cardiomyopathy (HCM)	-1.33	0.0253
KEGG_RIBOSOME	Ribosome	-1.25	0.0452
KEGG_CHEMOKINE_SIGNALING_PATHWAY	Chemokine signaling pathway	-1.25	0.0238
KEGG_CYTOKINE_CYTOKINE_RECEPTOR_INTERACTION	Cytokine-cytokine receptor interaction	-1.16	0.0204

Abbreviations: GSEA, gene set enrichment analysis; NES, normalized enrichment score.

Table S6. The ANCOM analysis of gut microbiota at genus level.

	W[1]	detected_0.9	detected_0.8	detected_0.7	detected_0.6
norank_f__Desulfovibrionaceae	105	TRUE	TRUE	TRUE	TRUE
Mucispirillum	103	TRUE	TRUE	TRUE	TRUE
Rikenellaceae_RC9_gut_group	103	TRUE	TRUE	TRUE	TRUE

[1] W value represents the significance of intergroup differences of the species.

Table S7. Abundance of gut microbial strains identified by LEFSe and ANCOM analysis at the genus level.

Characteristic bacteria	Control group	UVB group	EU group
Mucispirillum	14.7 ± 19.8	1389.0 ± 859.0	1183.0 ± 643.9
Rikenellaceae_RC9_gut_group	0.0 ± 0.0	556.3 ± 531.1	294.7 ± 559.3
norank_f__Desulfovibrionaceae	0.0 ± 0.0	255.3 ± 47.85	533.0 ± 239.0

Data was presented as mean ± SEM, $n = 6$.

Table S8. Global network parameters.

Parameters	Control group	UVB group	EU+UVB group
Node number	98	105	100
Edge number	403	383	381
Clustering coefficient	0.500	0.497	0.487
Characteristic path length	2.906	3.025	3.011
Average degree	9.413	8.424	8.842
Graph density	0.127	0.100	0.118

Table S9. Description of taxa information for each node in the control group network.

Name	Degree
g__Family_XIII_UCG-001	20
g__A2	20
g__Oscillibacter	18
g__Harryflintia	18
g__norank_f__Oscillospiraceae	18
g__Tuzzerella	18
g__norank_f__Ruminococcaceae	18
g__unclassified_f__Oscillospiraceae	17
g__Erysipelatoclostridium	17
g__Colidextribacter	17
g__Lachnospiraceae_FCS020_group	16
g__NK4A214_group	16
g__Anaerovorax	16
g__Parabacteroides	16
g__ASF356	16
g__Ralstonia	15
g__norank_f__Eubacterium_coprostanoligenes_group	15
g__Eubacterium_ventriosum_group	15
g__norank_f__Muribaculaceae	12
g__unclassified_f__Lachnospiraceae	12
g__Alloprevotella	12
g__Eubacterium_brachy_group	12
g__Intestinimonas	11
g__Eubacterium_oxidoreducens_group	11
g__Desulfovibrio	11
g__unclassified_k__norank_d__Bacteria	11
g__Lactococcus	11
g__unclassified_f__Atopobiaceae	10
g__Enterococcus	10
g__norank_f__Lachnospiraceae	10
g__norank_f__Clostridium_methylpentosum_group	10
g__unclassified_o__Oscillospirales	10
g__unclassified_o__Bacteroidales	10
g__Leuconostoc	10
g__Bacteroides	9
g__unclassified_c__Clostridia	9
g__Globicatella	9
g__Negativibacillus	9
g__Novosphingobium	8
g__Acetatifactor	8
g__Parvibacter	8

g__UCG-009	8
g__unclassified_f__Erysipelatoclostridiaceae	8
g__unclassified_o__Erysipelotrichales	8
g__norank_f__Caulobacteraceae	8
g__Marvinbryantia	8
g__Acinetobacter	8
g__Enterorhabdus	8
g__Lachnospiraceae_UCG-004	8
g__unclassified_p__Firmicutes	8
g__Lactobacillus	8
g__Bacillus	8
g__Lachnospiraceae_NK4A136_group	8
g__unclassified_c__Bacilli	8
g__Streptococcus	8
g__unclassified_f__Eggerthellaceae	8
g__Bifidobacterium	7
g__Lachnoclostridium	7
g__Mucispirillum	7
g__Turicibacter	6
g__Akkermansia	6
g__Escherichia-Shigella	6
g__Alistipes	6
g__norank_f__norank_o__Rhodospirillales	5
g__Eubacterium_fissicatena_group	5
g__norank_f__Peptococcaceae	5
g__GCA-900066575	5
g__Peptococcus	5
g__Candidatus_Saccharimonas	5
g__unclassified_f__Ruminococcaceae	5
g__Flavonifractor	4
g__Burkholderia-Caballeronia-Paraburkholderia	4
g__Butyricicoccus	4
g__Anaerotruncus	4
g__Helicobacter	4
g__Jeotgalicoccus	3
g__Eubacterium_hallii_group	3
g__Eubacterium_xylanophilum_group	3
g__Muribaculum	3
g__norank_f__norank_o__Clostridia_vadinBB60_group	3
g__Staphylococcus	3
g__Dubosiella	3
g__unclassified_c__Bacteroidia	2
g__unclassified_o__Burkholderiales	2
g__unclassified_f__Rikenellaceae	2

g__Ruminococcus_torques_group	2
g__Blautia	2
g__Bilophila	2
g__Parasutterella	2
g__Legionella	2
g__Macrococcus	2
g__Clostridium_sensu_stricto_1	2
g__Eubacterium_nodatum_group	1
g__norank_f__norank_o__Clostridia_UCG-014	1
g__Roseburia	1
g__Lachnospiraceae_UCG-006	1
g__Anoxybacillus	1
g__unclassified_f__Erysipelotrichaceae	1

Table S10. Description of taxa information for each node in the UVB group network.

Name	Degree
g__unclassified_o__Lactobacillales	22
g__Alistipes	17
g__Globicatella	14
g__Lachnospiraceae_UCG-010	14
g__Anoxybacillus	13
g__Lachnospiraceae_FCS020_group	13
g__norank_f__norank_o__Clostridia_vadinBB60_group	13
g__unclassified_f__Atopobiaceae	13
g__Candidatus_Saccharimonas	13
g__unclassified_f__Erysipelotrichaceae	13
g__unclassified_c__Bacteroidia	13
g__Erysipelatoclostridium	13
g__unclassified_o__Bacteroidales	13
g__Enterorhabdus	13
g__Intestinimonas	13
g__Butyricimonas	13
g__Lachnoclostridium	12
g__Lachnospiraceae_NK4A136_group	12
g__Oscillibacter	12
g__Eubacterium_oxidoreducens_group	12
g__Turicibacter	11
g__norank_f__Desulfovibrionaceae	11
g__Anaerotruncus	11
g__Lactococcus	11
g__GCA-900066575	11
g__Roseburia	11
g__Alloprevotella	11
g__norank_f__Muribaculaceae	11
g__Rikenellaceae_RC9_gut_group	11
g__Eubacterium_xylanophilum_group	10
g__Tuzzerella	10
g__Bilophila	10
g__norank_f__Peptococcaceae	10
g__norank_f__Lachnospiraceae	10
g__norank_f__Oscillospiraceae	9
g__Family_XIII_UCG-001	9
g__norank_f__Christensenellaceae	9
g__norank_f__Ruminococcaceae	9
g__unclassified_f__Oscillospiraceae	9
g__Bacteroides	9
g__Eubacterium_brachy_group	9

g__Butyricicoccus	8
g__Enterococcus	8
g__Bacillus	8
g__Family_XIII_AD3011_group	8
g__Parabacteroides	8
g__unclassified_f__Ruminococcaceae	8
g__Lachnospiraceae_UCG-006	8
g__norank_f__norank_o__Rhodospirillales	8
g__norank_f__UCG-010	8
g__Lactobacillus	7
g__unclassified_p__Firmicutes	7
g__Helicobacter	7
g__unclassified_k__norank_d__Bacteria	7
g__unclassified_f__Enterobacteriaceae	7
g__Desulfovibrio	7
g__unclassified_o__Oscillospirales	7
g__unclassified_f__Eggerthellaceae	7
g__Akkermansia	7
g__Faecalibaculum	6
g__Colidextribacter	6
g__A2	6
g__Rikenella	5
g__Parasutterella	5
g__Ralstonia	5
g__Bifidobacterium	5
g__Escherichia-Shigella	5
g__Negativibacillus	5
g__Leuconostoc	5
g__Mucispirillum	5
g__Flavonifractor	4
g__Harryflintia	4
g__Blautia	4
g__Anaerovorax	4
g__unclassified_f__Streptococcaceae	3
g__Weissella	3
g__unclassified_f__Rikenellaceae	3
g__Haloimpatiens	3
g__norank_f__Clostridium_methylpentosum_group	3
g__Candidatus_Soleaferrea	3
g__unclassified_f__Erysipelatoclostridiaceae	3
g__Corynebacterium	3
g__Dubosiella	3
g__Peptococcus	3
g__Candidatus_Arthromitus	3

g__NK4A214_group	3
g__Eubacterium_ventriosum_group	3
g__Burkholderia-Caballeronia-Paraburkholderia	3
g__unclassified_f__Lachnospiraceae	3
g__ASF356	3
g__Lachnospiraceae_UCG-004	3
g__Ruminococcus_torques_group	2
g__unclassified_f__Desulfovibrionaceae	2
g__unclassified_f__Prevotellaceae	2
g__unclassified_c__Bacilli	2
g__Streptococcus	2
g__Parvibacter	2
g__norank_f__norank_o__Saccharimonadales	2
g__Acetatifactor	2
g__Marvinbryantia	2
g__norank_f__Caulobacteraceae	1
g__Novosphingobium	1
g__Clostridium_sensu_stricto_1	1
g__Coriobacteriaceae_UCG-002	1
g__Eubacterium_nodatum_group	1

Table S11. Description of taxa information for each node in the EU+UVB group network.

Name	Degree
g__Lachnospiraceae_FCS020_group	22
g__Leuconostoc	19
g__Corynebacterium	19
g__Ruminococcus_torques_group	19
g__unclassified_f__Erysipelatoclostridiaceae	19
g__Mucispirillum	18
g__norank_f__Oscillospiraceae	18
g__Desulfovibrio	17
g__unclassified_o__Bacteroidales	16
g__Ralstonia	15
g__Anoxybacillus	14
g__norank_f__Muribaculaceae	14
g__Enterorhabdus	14
g__Alistipes	14
g__Enterococcus	14
g__Clostridioides	14
g__Akkermansia	14
g__Rikenellaceae_RC9_gut_group	14
g__Lactococcus	14
g__Alloprevotella	13
g__Lachnospiraceae_NK4A136_group	12
g__unclassified_f__Oscillospiraceae	12
g__Kurthia	12
g__unclassified_f__Microbacteriaceae	12
g__Burkholderia-Caballeronia-Paraburkholderia	12
g__Lachnospiraceae_UCG-006	11
g__unclassified_c__Bacilli	11
g__Erysipelatoclostridium	10
g__Globicatella	10
g__Flavonifractor	10
g__Anaerotruncus	9
g__Oscillibacter	9
g__Lactobacillus	9
g__norank_f__Ruminococcaceae	9
g__Blautia	9
g__Clostridium_sensu_stricto_1	9
g__Streptococcus	9
g__unclassified_f__Eggerthellaceae	8
g__GCA-900066575	8
g__norank_f__Peptococcaceae	8
g__ASF356	8

g__Butyricicoccus	8
g__Novosphingobium	7
g__Eubacterium_xylanophilum_group	7
g__unclassified_c__Clostridia	6
g__Intestinimonas	6
g__Negativibacillus	6
g__Escherichia-Shigella	6
g__Harryflintia	6
g__Turicibacter	6
g__Legionella	6
g__Family_XIII_UCG-001	6
g__A2	6
g__Tyzzerella	6
g__Eubacterium_oxidoreducens_group	6
g__Nocardioides	6
g__norank_f__Lachnospiraceae	5
g__norank_f__Clostridium_methylpentosum_group	5
g__Eubacterium_nodatum_group	5
g__unclassified_f__Atopobiaceae	5
g__Candidatus_Saccharimonas	5
g__norank_f__Caulobacteraceae	5
g__Tuzzerella	5
g__Dubosiella	5
g__unclassified_f__Lachnospiraceae	5
g__unclassified_p__Firmicutes	4
g__Colidextribacter	4
g__unclassified_f__Ruminococcaceae	4
g__Lachnoclostridium	4
g__norank_f__norank_o__Chloroplast	4
g__norank_f__Mitochondria	4
g__Exiguobacterium	4
g__Acinetobacter	4
g__Faecalitalea	4
g__Staphylococcus	3
g__Bifidobacterium	3
g__unclassified_o__Oscillospirales	3
g__norank_f__Desulfovibrionaceae	3
g__unclassified_f__Erysipelotrichaceae	3
g__Parabacteroides	3
g__norank_f__Obscuribacteraceae	3
g__Candidatus_Soleaferrea	3
g__Eubacterium_fissicatena_group	3
g__Arthrobacter	3
g__norank_f__UCG-010	3

g__Faecalibaculum	3
g__norank_f__norank_o__Clostridia_vadinBB60_group	3
g__Weissella	3
g__Roseburia	2
g__Bilophila	2
g__Eubacterium_brachy_group	2
g__unclassified_k__norank_d__Bacteria	2
g__Rikenella	2
g__Bacillus	1
g__Bacteroides	1
g__Kocuria	1
g__Lachnospiraceae_UCG-004	1
g__Parasutterella	1
g__Sphingomonas	1
g__Paracoccus	1
